# Uncovering the Important Genetic Factors for Growth during Cefotaxime-Gentamicin Combination Treatment in *bla*_CTX-M-1_ Encoding *Escherichia coli*

**DOI:** 10.3390/antibiotics12060993

**Published:** 2023-06-01

**Authors:** Mosaed Saleh A. Alobaidallah, Vanesa García, Richard De Mets, Sandra M. Wellner, Line E. Thomsen, Ana Herrero-Fresno, John Elmerdahl Olsen

**Affiliations:** 1 Department of Veterinary and Animal Sciences, Faculty of Health and Medical Sciences, University of Copenhagen, 1870 Frederiksberg, Denmark; alobaidallah@sund.ku.dk (M.S.A.A.); vanesag.menendez@usc.es (V.G.); sandra.wellner@sund.ku.dk (S.M.W.); leth@sund.ku.dk (L.E.T.); ahefr@food.dtu.dk (A.H.-F.); 2 Department of Clinical Laboratory Sciences, College of Applied Medical Sciences, King Saud bin Abdulaziz University for Health Sciences, Jeddah 21423, Saudi Arabia; 3 King Abdullah International Medical Research Center, Jeddah 22384, Saudi Arabia; 4 Laboratorio de Referencia de Escherichia coli (LREC), Departamento de Microbioloxía e Parasitoloxía, Facultade de Veterinaria, Universidade de Santiago de Compostela (USC), 27002 Lugo, Spain; 5 Department of Biomedical Sciences, Core Facility for Integrated Microscopy, University of Copenhagen, 2200 Copenhagen, Denmark; richard.demets@sund.ku.dk; 6 National Food Institute, Technical University of Denmark, 2800 Lyngby, Denmark

**Keywords:** *Escherichia coli*, cefotaxime, gentamicin, antibiotic resistance, extended-spectrum beta-lactamase, combination treatment, Transposon Directed Insertion-site Sequencing, helper drugs

## Abstract

Due to the rapid spread of CTX-M type ESBLs, the rate of resistance to third-generation cephalosporin has increased among Gram-negative bacteria, especially in *Escherichia coli*, and there is a need to find ways to re-sensitize ESBL *E. coli* to cephalosporin treatment. A previous study showed that genes involved in protein synthesis were significantly up-regulated in the presence of subinhibitory concentration of cefotaxime (CTX) in a CTX-M-1-producing *E. coli*. In this study, the interaction between CTX and gentamicin (GEN), targeting protein synthesis, was evaluated in MG1655/pTF2, and the MIC of CTX was strongly reduced (128-fold) in the presence of this combnation therapy. Since the underlying mechanism behind this synergy is not known, we constructed a saturated transposon mutant library in MG1655/pTF2::*bla*_CTX-M-1_ containing 315,925 unique transposon insertions to measure mutant depletion upon exposure to CTX, GEN, and combination treatment of CTX and GEN by Transposon Directed Insertion-site Sequencing (TraDIS). We identified 57 genes that were depleted (log_2_FC ≤ −2 and with q.value ≤ 0.01) during exposure to CTX, 18 for GEN, and 31 for combination treatment of CTX and GEN. For validation, we deleted eight genes that were either uniquely identified in combination treatment, overlapped with monotherapy of GEN, or were shared between combination treatment and monotherapy with CTX and GEN. Of these genes, we found that the inactivation of *dnaK*, *mnmA*, *rsgA*, and *ybeD* increased the efficacy of both CTX and GEN treatment, the inactivation of *cpxR* and *yafN* increased the efficacy of only CTX, and the inactivation of *mnmA*, *rsgA*, and *ybeD* resulted in increased synergy between CTX and GEN. Thus, the study points to putative targets for helper drugs that can restore susceptibility to these important drugs, and it indicates that genes involved in protein synthesis are essential for the synergy between these two drugs. In summary, the study identified mutants that sensitize ESBL-producing *E. coli* to CTX and a combination of CTX and GEN, and it increased our understanding of the mechanism behind synergy between β-lactam and aminoglycoside drugs. This forms a framework for developing new strategies to combat infections caused by resistant bacteria.

## 1. Introduction

Drug-resistant infections account for the death of around 700,000 people globally every year, and this number is estimated to grow to more than 10 million and cost the global economy up to 100 trillion USD by 2050 if the problem is not solved [1]. The World health organization (WHO) has published a list of resistant bacteria, which should be prioritized when searching for novel antibiotics and *Enterobacteriaceae* with resistance to third generation cephalosporins are among the top priorities on this list [2].

β-lactam antibiotics are bactericidal agents that inhibit peptidoglycan synthesis by binding to Penicillin-binding proteins (PBPs), thereby preventing bacterial cell wall synthesis. Their high use has caused widespread resistance among Gram-negative bacteria. A particular concern is Extended Spectrum β-lactamase (ESBL) producing *E. coli*, which are capable of hydrolyzing the β-lactam ring in third and fourth generation cephalosporins [3]. ESBL genes are primarily carried on plasmids and include enzymes of the TEM, SHV, and CTX-M classes, and among these classes, the broadest dissemination has been detected for the CTX-M family [3,4,5]. To date, more than 190 variants of CTX-M-β-lactamases have been identified [6]. They are grouped into five gene families (CTX-M-1, CTX-M-2, CTX-M-8, CTX-M-9, and CTX-M-25) [7,8].

One of the possible strategies to overcome antimicrobial-resistant organisms is to use combination treatment, especially with drugs that have synergistic interaction [9,10]. Another possibility is to revitalize the existing antimicrobials by identifying helper drugs that can inhibit the resistant bacteria from growing at full capacity while expressing their resistance mechanisms [11]. A recent study characterized the global transcriptomic response in a CTX-M-1-producing *E. coli* growing in the presence of subinhibitory concentrations of the third-generation cephalosporin, cefotaxime (CTX) and found that a high number of genes, which were unrelated to the resistance mechanism, were significantly regulated [12]. The study demonstrated that genes involved in protein synthesis were enriched among the up-regulated genes, and in accordance with this, combination treatment with low concentrations of chloramphenicol (CHL), which inhibits protein synthesis, significantly reduced the MIC of CTX [12]. Since CHL is rarely used for systemic treatment due to its hematologic toxicity [13], the combination of CTX with aminoglycosides, such as gentamicin (GEN), is one of the most commonly prescribed combination treatments in clinical practice [14,15]. However, the molecular mechanism behind how these combination treatments work together remains surprisingly elusive.

There is no direct link between regulation and essentiality of genes, and thus it may be cumbersome to identify the most promising targets to re-sensitize ESBL bacteria to treatment from transcriptomic data. Jana et al. [11] demonstrated a more suited approach relying on libraries of random transposon mutants in their study of the secondary resistome (SR) of *Klebsiella pneumoniae* toward colistin, and in the current study, we have used this approach to investigate the SR of ESBL *E. coli* towards CTX. To increase the understanding of the synergistic effect between CXT and GEN, we further determined the SR to GEN treatment in the ESBL strain as well as the SR to the combination treatment. Thus, the aims of the study were to assign fitness values to all non-essential genes in the CTX-M-1-producing *E. coli* strain for the ability to grow in the presence of ½ MIC of CTX, GEN, and combination of CTX with GEN, and to investigate whether inactivation of genes with a high fitness effect would re-sensitise the bacteria to the drugs.

## 2. Materials and Methods

### 2.1. Bacterial Strains

Bacterial strains and plasmids used in this study are listed in Table S1. The main strain used was *E. coli* MG1655 carrying a *bla*_CTX-M-1_ gene on the IncI1 plasmid pFT2 [16]. Strains were grown in Difco™ lysogeny broth (LB), Lennox (Becton, Dickinson, Albertslund, Denmark), and on LB agar plates (Becton, Dickinson, Albertslund, Denmark) overnight at 37 °C, except for the strains harboring the temperature-sensitive plasmid, pKD46, which were grown at 30 °C. Where needed, media were supplemented with antibiotics (Sigma, Copenhagen, Denmark), including 20 mg/L CTX, 50 mg/L kanamycin (Kan), 20 mg/L gentamicin (GEN).

### 2.2. Determination of MIC and Interaction between CTX and GEN

The minimum inhibitory concentrations (MICs) of CTX, GEN, and other generation cephalosporins, including cefazolin (CFZ), cefoxitin (FOX), ceftazidime (CAZ), cefepime (FEP), and ceftaroline (CPT) against the MG1655/pTF2 and its mutant-derivatives strains were determined by broth microdilution method following CLSI guidelines [17]. The interaction between CTX and GEN was determined by the chequerboard assay as described [18,19] with some modifications. Briefly, CTX and GEN (Sigma, Copenhagen, Denmark) were used in the ranges 0–512 and 0–4 mg/L, respectively. The first antibiotic of the combination was two-fold serially diluted in the horizontal line of the panel, while the second antibiotic was serially diluted in the vertical line of the panel. The inoculum for each strain was prepared by using a McFarland 0.5 standard (1–2 × 10^8^ CFU/mL) and a Sensititre Nephelometer (Thermo Scientific^TM^, Roskilde, Denmark). All the wells containing 100 μL of Mueller–Hinton broth II (MHB-II) (Sigma, Copenhagen, Denmark) were inoculated with 100 μL of inoculum to a final cell density of around 5 × 10^5^ CFU/mL and incubated for 18–22 h. The fractional inhibitory concentration (FIC) and FIC index (FICI) were calculated using the formula: FIC of drug A = MIC of drug A in combination/MIC of drug A alone, FIC of drug B = MIC of drug B in combination/MIC of drug B alone, and FICI = FIC of drug A+ FIC of drug B [20]. The interpretation of synergism was defined as an FICI ≤ 0.5, additive or indifference was defined as an FICI > 0.5–1 or >1–4, respectively, and antagonism was defined as an FICI > 4 [21,22]. The values were obtained from triplicate biological replicates per condition.

### 2.3. TraDIS Library Construction and Validation

Electrocompetent cells of *E. coli* MG1655/pTF2 were made as previously described [23,24,25]. Briefly, the bacterial cells were grown overnight in LB medium, then 1:100 diluted in sterilized LB and grown to an OD_600_ of 0.5–0.6. The cells were then centrifuged and washed in 1 × volume of cold Milli-Q water, 0.5 × volume of cold Milli-Q water, 0.05 × volume of 10% cold glycerol, respectively. After washing, the cells were resuspended in 0.002 × volume of 10% cold glycerol, and aliquots of 60 μL electrocompetent cells were stored at −80 °C until use. For electroporation, a 60 μL electrocompetent cells aliquot was mixed with 1 μL EZ-Tn5 < KAN-2 > Tnp Transposome (Epicentre) and electroporated in a 1 mm cuvette using a BioRad MicroPulser set to 1.8 kV. The mixture was immediately resuspended in 1 mL Super Optimal broth with Catabolites repression (SOC) medium and incubated at 37 °C for 2 h. Then, 100 μL of aliquots was spread on nine large LB agar plates, 15 cm, supplemented with 50 mg/L Kan. After incubation overnight at 37 °C, the kanamycin-resistant colonies from each plate were resuspended in 2–3 mL sterilized LB supplemented with glycerol 20% using a sterilized bacteriological spreader and transferred into 50 mL tube and stored at −80 °C. Each tube contained an estimated 10,000–50,000 mutants. The final input library contained 3 × 10^5^ mutants that were generated by pooling 13 mutant batches, resulting in a cell suspension of 1.3 × 10^11^ CFU/mL.

### 2.4. TraDIS Library Antimicrobial Exposure and Sequencing

**Figure 1:**
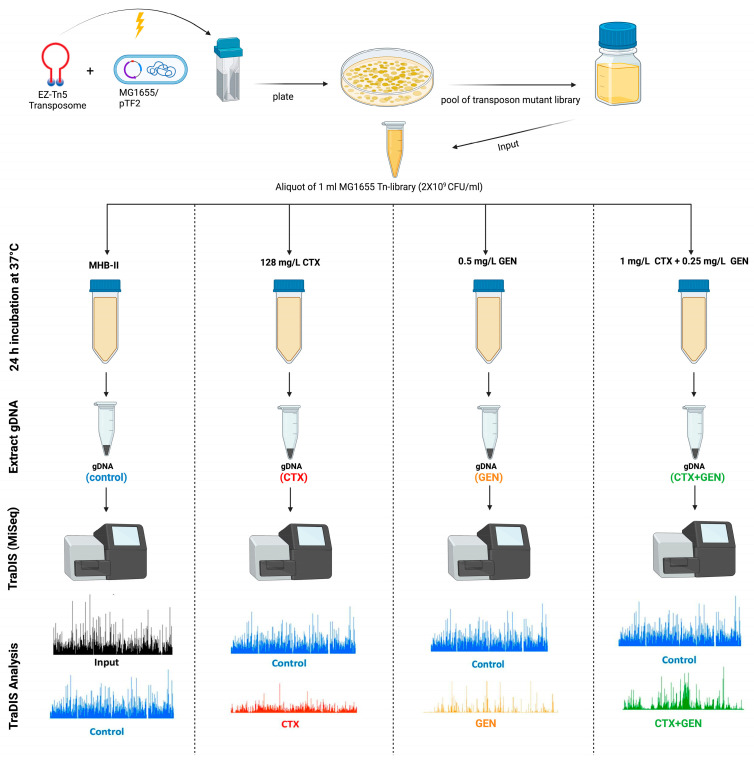
Design of the genetic screening to identify the SR in an ESBL *E. coli* to treatment with CTX, GEN, and combination treatment with these two drugs. A transposon mutant library was constructed in MG1655/pTF2 (input) and incubated in four different conditions, without antibiotic (control), with 128 mg/L CTX or 0.5 mg/L GEN, or 1 mg/L CTX with 0.25 mg/L GEN for 24 h. The cells were harvested, genomic DNA for each condition (output) was extracted and analyzed using Bio::TraDIS pipeline.

In duplicate, 100 μL of 1 mL aliquots of the input library, containing approximately 2 × 10^9^ mutants, were diluted into four falcon tubes containing 9.9 mL MHB-II, 9.9 mL MHB-II supplemented with 128 mg/L CTX, 9.9 mL, MHB-II supplemented with 0.5 mg/L GEN, and 9.9 mL MHB-II supplemented with 1 mg/L CTX plus 0.25 mg/L GEN, corresponding to ½ MIC of each treatment (Figure 1). After 24 h of incubation at 37 °C, 1 mL from each culture (output library) was centrifuged at 12000 rpm for 2 min, and the resulting pellet was used for DNA isolation using GenElute™ Bacterial Genomic DNA kit (Sigma-Aldrich, Soeborg, Denmark) following manufacturer’s instructions. In addition, 500 μL from the 1 mL aliquot of the input library, containing approximately 2 × 10^9^ mutants, was also used for DNA extraction. The quality of DNA was evaluated by NanoDrop (Thermo Fisher Scientific, Roskilde, Denmark). A ratio of OD260/OD280 between 1.8 and 2.0 and a ratio of OD260/OD230 between 2.0 and 2.2 were set as criteria for samples to be used for sequencing (Table S2). The DNA concentration was measured using Qubit dsDNA HS Assay Kit (Thermo Fisher) (Table S2). For TraDIS sequencing, 2–4 μg of DNA was fragmented using Covaris M220 (Covaris, Woburn, MA, USA) into approximately 300 bp fragments, and each library was prepared for sequencing following the protocols previously described [25,26], using primers (Table S3) specific for PCR enrichment of the DNA fragments tagged with transposon. PCR amplified fragment libraries were then pooled and sequenced on a MiSeq instrument using a MiSeq reagent kit V2 (50 cycles) (Illumina, San Diego, CA, USA) following the recipe previously described [26].

### 2.5. Analysis of TraDIS Data

The analysis of sequences containing the transposon was performed as described [26] using the Bio::TraDIS pipeline (https://github.com/sanger-pathogens/Bio-Tradis, accessed on 26 June 2022) with some modifications previously described [25]. Briefly, the processing of raw reads was performed using *fq2bam.pl* script, which relocates the tag from the read name to the front of the read and converts the files to SAM format. Then, the generated SAM files were converted to BAM format by using samtools. The generated BAM format files were then used for analyses with *check_tradis_tags* and *add_tradis_tags* scripts. Afterwards, the output BAM files were converted to FASTQ format using samtools. Next, the *bacteria_tradis* mapping pipeline was used, and the obtained FASTQ files were filtered and trimmed for ten bases matching the 3′ end of the transposon. The generated files were then mapped against the MG1655 reference genome (GenBank:LR881938) using SMALT short-read mapper (https://www.sanger.ac.uk/tool/smalt-0/, accessed on 26 June 2022). After mapping, the precise transposon insertion site was assessed, and unique insertion sites (UISs) and read counts were determined per gene. Artemis version 18.1.0 [27] was used to visualize the UISs and read counts, and DNAPlotter version 18.1.0 [28] was used to create the circular genome diagram. Subsequent analysis steps were done using R scripts (*tradis_essentiality.R* and *tradis_comparisons.R*) included in the Bio::Tradis pipeline to assess the log_2_ fold change (log_2_FC) of read counts and q.value for each gene between test and control samples. To identify the important genes for survival in presence of antimicrobials, the significant genes were defined as genes with a log_2_FC ≤ −2 and q.value ≤ 0.01. The complete output from R scripts showing the log_2_FC and q.value of each gene appears in Table S4. The TraDIS raw reads of this study were deposited on the European Nucleotide Archive (ENA) under accession number (PRJEB52919). STRING (https://www.string-db.org, accessed on 30 June 2022) analysis was used to detect interactions between genes where mutation caused ≤ −2 log_2_FC between the challenge and control condition and to highlight the enrichment of KEGG terms and Gene Ontology (GO) terms based on these genes.

### 2.6. Deletion of Selected Genes in MG1655/pTF2

Mutants in MG1655/pTF2 were constructed by Lambda Red recombination system using the plasmids pKD46 and pKD4, essentially following the protocols described [29,30]. Correct insertion of the antibiotic gene cassettes in the target genes was confirmed by PCR. Primers used for gene knockout and for PCR control of gene insertion are listed in Table S5.

### 2.7. Growth Experiments

Bacterial growth experiments were performed in two biological replicates with two technical replicates for each strain on Bioscreen C (Thermo Labsystems, Helsinki, Finland) for 24 h at 37 °C. Briefly, 250 μL of MHB-II was inoculated with cells from LB agar plates to a final cell density of approximately 10^6^ CFU/mL, using a Sensititre™ Nephelometer (Thermo Fisher Scientific, Roskilde, Denmark) with a 0.5 McFarland standard (1–2 × 10^8^ CFU/mL). The cultures were then grown without antibiotic or supplemented with either 128 mg/L CTX, 0.5 mg/L GEN, or a combination of CTX and GEN of 1 + 0.25 mg/L. The OD_600_ was measured every 20 min with continuous shaking, and the growth curves were obtained using GraphPad Prism 9 (GraphPad Software, San Diego, CA, USA).

### 2.8. Morphology of Bacteria

The WT strains MG1655/pTF2 and mutant strains (*ΔcpxR*, *ΔdnaK*, *ΔmnmA*, *ΔrsgA*, *ΔyafN*, and *ΔybeD)*, which were identified to reduce the MIC of CTX, were inoculated into LB. After overnight growth at 37 °C, the strains were inoculated into sterilized LB to a starting OD_600_ of 0.05 and further incubated at 37 °C with continuous shaking to reach an OD_600_ of 0.3–0.4. To minimize the overnight culture residues and ensure that most of the cells were in early exponential phase, the strains were re-inoculated into new LB to a starting OD_600_ of 0.05, followed by incubation at 37 °C with continuous shaking to reach an OD_600_ of 0.3. CTX was then added to the cultures to a final concentration of 128 mg/L, and the treated and not-treated cultures were incubated for an additional hour. A volume of 900 μL of each culture was mixed with 100 μL of 37% formalin, resulting in cells fixed with 3.7% formalin. Cells were then washed two times with 1 × DPBS and then 1:10 diluted in 1 × DPBS. A volume of 50 μL was spread on Nunc™ Glass Bottom Dishes (Thermo Scientific™) coated with poly-L-lysin solution (Sigma, Copenhagen, Denmark). Cells were observed by differential interference contrast (DIC) using Zeiss LSM 780 laser scanning confocal microscope equipped with AxioCam camera. Bacteria segmentation was performed by training a custom Cellpose [31] model using cyto2 as a base model. The custom model was trained on 12 images corrected manually to segment correctly bacteria of various sizes and bending. The predictions were then exported into text files and converted into ROI in Fiji to measure the Feret diameter and extract the bacteria length. The experiment was repeated two times per condition, and no intravariability was noted during the analysis.

### 2.9. Time-Kill Assays

The activity of CTX against WT and its mutant-derivatives strains, which were identified to increase susceptibility to CTX, was evaluated by measuring the reduction of CFU/mL in the presence of antibiotic. Briefly, 10 mL MHB-II containing 10^6^ CFU/mL for each strain was prepared as described above, and 128 mg/L CTX was added to each tube and incubated at 37 °C for 24 h with continuous shaking. The CFU/mL was determined after several time points (4, 8, and 24 h). Two independent biological replicates were performed, and the time-kill curves for WT against each mutant strain were depicted using GraphPad Prism 9.

### 2.10. RNA Extraction and RT-qPCR

Single colonies of the WT and mutant strains that enhance the efficacy of CTX were grown separately in MHB-II with continuous shaking overnight at 37 °C. The cultures were then diluted to a starting OD_600_ of 0.05 and grown with different concentrations of CTX, corresponding to ½ MIC of the strains, to OD_600_ of 0.5–0.6. Total RNA for each sample was extracted by mechanical disruption with a FastPrep-24 homogenizer (MP Biomedicals, Solon, OH, USA) and RNeasy Mini Kit (Qiagen, Sollentuna, Sweden). The quality and quantity of extracted RNA was evaluated by NanoDrop. 600 ng of the RNA samples were purified by DNA digestion using TURBO™ DNase (2 U/μL) (Ambion^®^, Naerum, Denmark), and the treated RNA was reverse transcribed into cDNA using the High-Capacity cDNA Reverse Transcription Kit (Life Technologies, Naerum, Denmark). Reverse-transcribed-quantitative real time polymerase chain reaction (RT-qPCR) was performed using FastStart Essential DNA Green Master (Roche, Hvidovre, Denmark) and a LightCycler 96 (Roche, Hvidovre, Denmark). The expression of *bla*_CTX-M-1_ in mutant strains was calculated compared to expression in the WT strain in the presence of ½ MIC CTX. The expression data were normalized to a validated reference gene *gapA* [16]. The results were calculated by 2^−ΔΔCT^ method [32]. Two independent biological replicates were performed using two technical replicates. Primers are listed in Table S5.

### 2.11. In Silico Homology Study

To evaluate whether the targets that increased susceptibility to CTX upon deletion share similarity to host proteome, the protein sequences of CpxR, YafN, DnaK, MnmA, RsgA, and YbeD were obtained from Artemis, and the sequence homology to human proteins was searched using NCBI *Homo sapiens* Protein BLAST (BLASTp) (http://blast.ncbi.nlm.nih.gov/Blast.cgi, accessed on 16 February 2023). The proteins that showed no hits at the E-value cutoff of 10 × 10^−10^ were considered non-homologous proteins [33].

### 2.12. Statistical Analysis

The Kruskal–Wallis rank sum test followed by Dunn’s test was used to recognize significant changes in bacterial cell length of mutants compared to the WT (control) in the absence and presence of CTX. Statistical analysis for time-kill assay was performed by comparing the mean log_10_ CFU/mL of WT and mutant strains using multiple *t*-tests. For qPCR, the significant changes in fold change of *bla*_CTX-M-1_ in the WT to the mutant strains in the presence of ½ MIC CTX was evaluated using one-way ANOVA. A *p*-value < 0.05 was considered significant.

## 3. Results

### 3.1. Validation of Synergy between CTX and GEN

The MIC of CTX and GEN for MG1655/pTF2 were 256 and 1 mg/L, respectively. The synergistic interaction between CTX and GEN was identified by chequerboard assays, and the MIC of CTX was found to be reduced significantly from 256 to 2 mg/L (128-fold reduction) in the presence of 0.5 mg/L GEN with an FICI of 0.5 (Figure S1). Therefore, a combination of 1 mg/L CTX and 0.25 mg/L (1/2 of the synergy concentration) was used as treatment concentrations for the transposon library to elucidate the mechanism of synergy.

### 3.2. Generation of a High-Density Mutant Library and Sequencing Using TraDIS Protocol

A high-density Tn5 mutant input library was constructed in MG1655/pTF2, and the library of Kan^R^ mutants was exposed to three antimicrobial selection conditions (Figure 1). The reads of input and output libraries were mapped to K-12 MG1655 reference genome, with the percentage of mapped reads ranging from 84.38% to 92.03% (Table 1). When merging the duplicates of the input library, the saturation of Tn5 insertions across the MG1655 genome consisted of 315,925 unique transposon insertions (Figure S2) with an average of one Tn5 insertion every 14.6 base pairs (Table 1). Furthermore, 367 genes were identified to be essential for growth on LB agar plates supplemented with kanamycin, judged from the absence of insertions in these genes in the input library (Table S6).

### 3.3. Identification of Genes Relevant for Growth with and without Antibiotics

**Figure 2:**
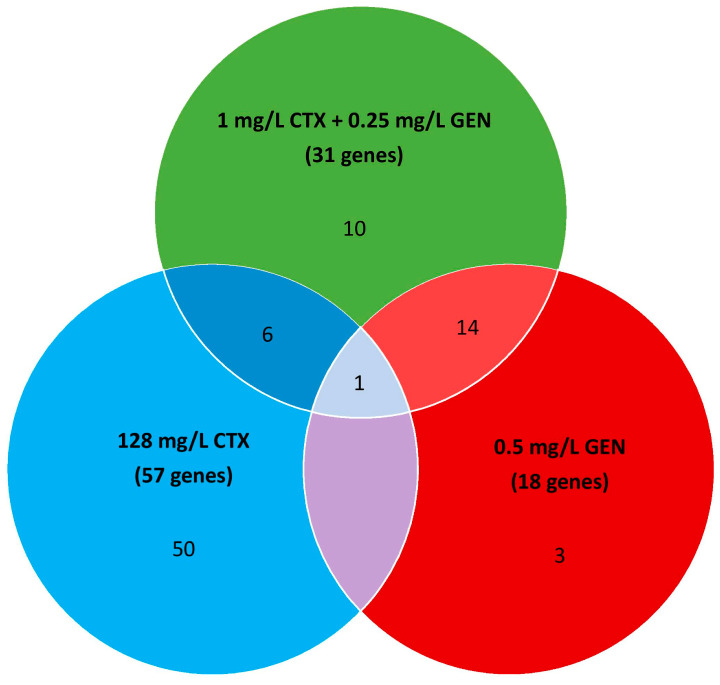
Venn diagram of the number of genes in the SR detected in MG1655/pTF2 upon exposure to ½ MIC concentrations of CTX, GEN, and combination treatment with CTX and GEN. Significantly depleted genes were defined as those showing Log_2_FC ≤ −2 with q.value ≤ 0.01.

The comparison of Tn5 insertions between the input library and the untreated library (control) resulted in the identification of 103 genes that were important for growth in MHB-II (Table S4), and comparison between the untreated library (control) and antibiotic-treated libraries identified 57, 18, and 31 genes which were important for growth with CTX, GEN, and CTX+GEN combination treatments, respectively (Figure 2, Table S4). Of the 31 genes identified for the combination treatment, 6 and 14 genes overlapped with genes significantly affected in the libraries exposed to monotherapy of CTX and GEN, respectively, and one gene (*cpxR*) was part of the SR in all three libraries. Thus, of the 31 genes identified as SR to combination treatment, only ten genes were unique to the library exposed to the combination treatment.

### 3.4. The Secondary Resistome to CTX

The top 20 genes, according to Log_2_FC after CTX treatment, including genes involved in many different cell systems (Table 2) and insertion of transposons into the gene *lpoB*, which is involved in peptidoglycan synthesis [34,35], caused the highest fitness defect (Log_2_FC = −10.10). To further understand the cellular responses, STRING analysis was performed based on genes showing Log_2_FC ≤ −2 and q.value ≤ 0.01. A graphic presentation of the interactions between the genes is given in Figure S3. The significantly affected genes corresponded to the enrichment of two KEGG pathways, the TCA cycle and carbon metabolism. Analysis of GO terms showed that 12 GO terms were significantly enriched in the biological process category, with cell cycle, cell division and bacteriocin transport having the highest scores. No terms were enriched in the category Molecular Functions, while 14 terms were significantly enriched in the category Cellular Components. The top enriched terms were cell division site, cellular anatomical entity, and cell envelope (Table S7).

### 3.5. The Secondary Resistome to GEN

The SR genes to GEN treatment in the CTX-resistant, GEN-sensitive MG1655/pTF2 are listed in Table 3. Genes belonging to the F_1_F_0_-ATP synthase subunits [36], such as *atpF*, *atpG*, *atpD*, *atpH*, *atpC*, *atpA*, and *atpB*, were among the highest attenuated genes following exposure to GEN treatment. STRING analysis was used to analyze interactions between the genes in the SR, and the encoded functions were elucidated by the enrichment of KEGG pathway and GO terms. A graphical illustration of the interactions between genes is shown in Figure S4. The significantly affected genes corresponded to the enrichment of three KEGG pathways, the oxidative phosphorylation pathway, metabolic pathways, and taurine and hypotaurine metabolism. Analysis of GO terms showed that 13 GO terms were enriched in the biological process category, among which purine ribonucleotide biosynthetic process, nucleobase-containing small molecule metabolic process, and carbohydrate derivative biosynthetic process showed the highest significant enrichment values. Proton-transporting ATP synthase activity, proton-transporting ATPase activity, inorganic molecular entity transmembrane transporter activity, and ion transmembrane transporter activity were the enriched GO terms for Molecular functions. Ten GO terms related to Cellular components were enriched after the treatment of MG1655/pTF2 with 0.5 mg/L GEN, with the proton-transporting ATP synthase complex, membrane protein complex, and protein-containing complex having the highest significant scores (Table S8).

### 3.6. The Secondary Resistome to CTX-GEN Combination Treatment

To elucidate the genes and, therefore, indirectly the mechanism(s) associated with the synergy between CTX and GEN, the transposon input library was grown in the presence of 1 mg/L CTX and 0.25 mg/L GEN, which corresponded to ½ MIC detected under synergy conditions according to the chequerboard assay results (Figure S1). Based on the TraDIS analysis, 10 genes out of the 31 genes identified in the combination treatment were unique to this condition, while one gene (*cpxR*), as mentioned, was shared between all libraries, and the remaining genes were shared with the CTX or GEN output libraries (Table 4). As for GEN treatment, most of the fitness genes during exposure to combination treatment belonged to the ATP synthase subunits. However, the common genes showed a higher fold change in the combination treatment than in the GEN treatment despite the lower concentrations of the two drugs when combined, except for the *atpA* gene (Table 4).

The graphical illustration of the interactions between genes in the SR to the combination treatment is indicated in Figure S5. The STRING analysis showed enrichment of only one KEGG pathway, the oxidative phosphorylation, which was also found for the GEN treatment. Fourteen GO terms for biological processes were enriched, with purine ribonucleotide biosynthetic process, bacteriocin transport, and protein import showing the highest significant scores. In addition to the four GO terms for Molecular functions that were enriched in GEN treatment, ligase activity was uniquely found as a term in the presence of combination treatment. Eight GO terms for Cellular components were enriched, of which six terms were also enriched after treatment with GEN (e.g., ATP synthase complex, membrane protein complex). In addition, the cell division site term was listed when the strain was treated with CTX alone. The term protein-containing complex was found to be enriched in all three libraries. GO terms such as cellular response to bacteriocin and organic substance, transport, tRNA wobble position uridine thiolation, and ligase activity were uniquely enriched in the combination treatment. The list of all terms enriched in combination treatment is shown in Table S9.

### 3.7. Validation of Genes Identified by TraDIS

Since one of the aims of the current study was to understand the synergistic effect of CTX and GEN in MG1655/pTF2, a total of eight fitness genes that were either uniquely identified in the library exposed to combination treatment, overlapped between the libraries exposed to GEN and combination treatment, or which were shared between all three libraries were individually knocked-out by site-directed mutagenesis to investigate their role for growth in the absence and presence of three antibiotic-related conditions. The functional classifications of these genes were transcription regulation (*cpxR*, *yafN)* [37,38], stress response (*ydeI* and *dnaK*) [39,40], translation, ribosomal structure and biogenesis (*mnmA* and *rsgA*) [41,42,43], protein transport (*yajC*) [44], and hypothetical protein (*ybeD*) [45].

**Figure 3:**
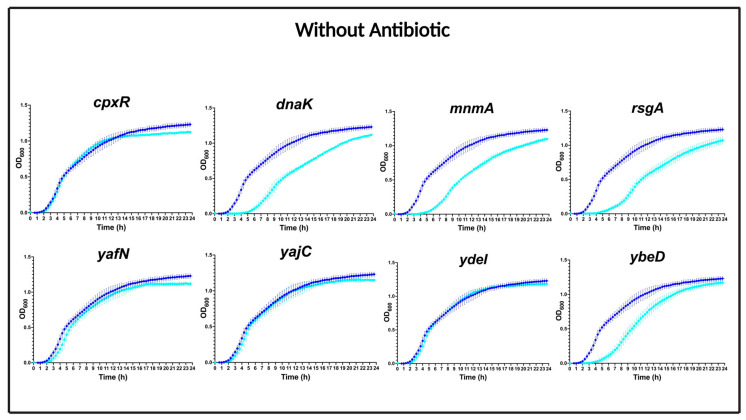
Growth curves of selected mutants in the absence of antibiotics. Growth of MG1655/pTF2 (blue line) compared to mutants (cyan lines) in MHB-II. Data were combined from two independent biological replicates with four technical replicates, presented as mean ± standard deviation.

The growth of mutant strains compared to the WT strain without the presence of antibiotics is shown in Figure 3. The analyses showed that the deletions of *cpxR*, *yafN*, *yajC*, and *ydeI* did not cause growth effects compared to the WT strain in the absence of antimicrobials. In contrast, the deletions of *dnaK*, *mnmA*, *rsgA*, and *ybeD* resulted in a slower growth of the mutants compared to the WT strain, mainly caused by slightly prolonging the lag phase.

**Figure 4:**
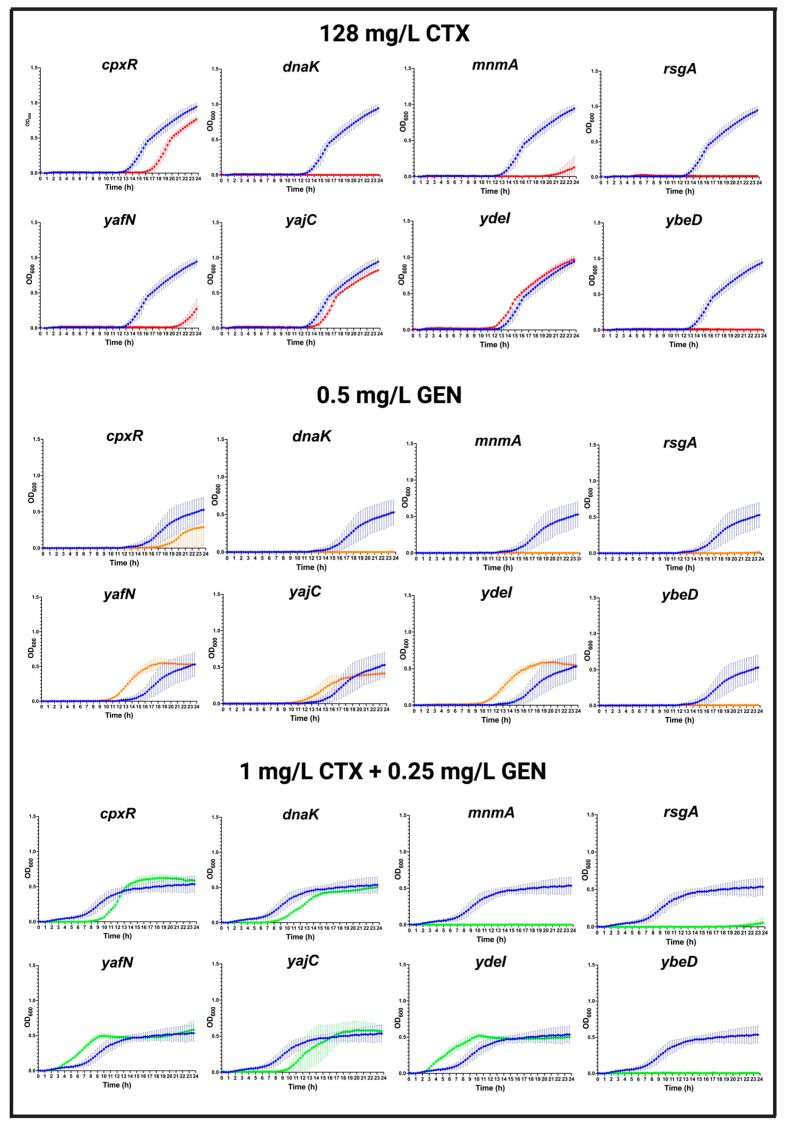
Growth curves of selected mutants in the presence of antibiotics. Growth of MG1655/pTF2 (blue line) compared to mutants (red, orange, or green lines) in MHB-II supplemented with (128 mg/L CTX, 0.5 mg/L GEN, and 1 mg/L CTX plus 0.25 mg/L GEN), respectively. Data were combined from two independent biological replicates with four technical replicates, presented as mean ± standard deviation.

In the presence of 128 mg/L CTX, only a slight growth defect was seen in the *cpxR* mutant compared to WT strain, whereas remarkable growth defects were found in *dnaK*, *rsgA*, *ybeD*, *mnmA*, and *yafN* mutant strains, respectively. No notable growth defects were found in *yajC* and *ydeI* mutant strains compared to the WT (Figure 4).

In the presence of 0.5 mg/L GEN, a minor growth defect was also seen in *cpxR* mutant strain compared to the WT. No growths were detected after the deletion of *dnaK*, *mnmA*, *rsgA*, and *ybeD* during 24 h. Surprisingly, the *yafN* and *ydeI* mutant strains were found to grow faster than the WT in the presence of 0.5 mg/L GEN. No apparent difference was observed in *yajC* mutant compared to the WT strain (Figure 4).

In the presence of the combination treatment of 1 mg/L CTX plus 0.25 mg/L GEN, the mutant strains *cpxR*, *dnaK*, and *yajC* were found to grow slightly slower than the WT strain. On the contrary, the deletions in *yafN* and *ydeI* were growing faster than the WT. No growths were identified in mutant strains with deletion of *mnmA*, *rsgA*, and *ybeD* in the presence of combination treatment (Figure 4).

Based on the results above, chequerboard assays were performed with mutants with deletion of *mnmA*, *rsgA*, and *ybeD* to determine whether these mutations were associated with increased synergy between CTX and GEN compared to the WT strain. Based on the chequerboard assays, the *mnmA* and *rsgA* mutant strains were found to be fully sensitive to CTX according to CLSI breakpoints [46], with a MIC of 0.5 mg/L in the presence of only 0.25 mg/L GEN with an FICI of 0.53. For the *ybeD* mutant strain, the strain was also susceptible to CTX with a MIC of 1 mg/L in the presence of only 0.125 mg/L GEN with an FICI of 0.56. Since these FICIs values were between 0.51–1, they were considered additive effects. However, synergy (FICI ≤ 0.5) was found between 4 mg/L CTX and 0.125 mg/L GEN, 2 mg/L CTX and 0.125 mg/L GEN, and 4 mg/L CTX and 0.06 mg/L GEN for *mnmA*, *rsgA* and *ybeD* mutant strains, respectively. Chequerboard assays with all FICIs are shown in Figure S6.

### 3.8. Increased Susceptibility to Antimicrobials

To investigate whether growth attenuation would lead to increased susceptibility of the mutant strains to treatment, MICs for mutant strains were obtained using broth microdilutions (Table 5). The mutations of *cpxR*, *dnaK*, *mnmA*, *rsgA*, *yafN*, and *ybeD* were found to decrease the MIC to CTX with 8-fold, 32-fold, 16-fold, 32-fold, 8-fold, and 16-fold, respectively, compared to the WT. In addition, the mutant strains of *dnaK*, *mnmA*, *rsgA*, and *ybeD* also showed increased susceptibility to GEN with 2-fold or 4-fold reduction of the MIC compared to the WT.

Since the deletions of *cpxR*, *dnaK*, *mnmA*, *rsgA*, *yafN*, and *ybeD* were found to reduce the MIC to CTX compared to the WT strain, we extended the analysis for these mutants by testing the MICs of five additional cephalosporins: CFZ (first generation), FOX (second generation), CAZ (third generation), FEP (fourth generation), and CPT (fifth generation). Mutations of *dnaK*, *mnmA*, and *rsgA* resulted in increased susceptibility to all five antibiotics (Table 5). Mutations of *cpxR*, *yafN*, and *ybeD* exhibited increased susceptibility to at least one cephalosporin antibiotic: Mutation of *cpxR* led to increased susceptibility to CFZ, mutation of *yafN* led to increased susceptibility to CFZ and FOX, and mutation of *ybeD* led to increased susceptibility to CFZ, FOX, CAZ, and FEP.

### 3.9. Determination of Filament Formation in the Mutant Strains

Many β-lactam antibiotics, including CTX, induce filament formation in susceptible Gram-negative bacteria [47,48]. This filamentation depends on the antibiotic concentration [47,49]. Therefore, a fixed concentration of CTX, which corresponds to ½ MIC of the WT, was used for WT strain and *cpxR*, *dnaK*, *mnmA*, *rsgA*, *yafN*, and *ybeD* mutant strains were identified to restore susceptibility to CTX. These strains were then evaluated for morphological changes using fixed-cell imaging. To ensure that the morphology changes were caused by the exposure to CTX, the microscopic appearance of the same strains was evaluated in the absence of CTX.

**Figure 5:**
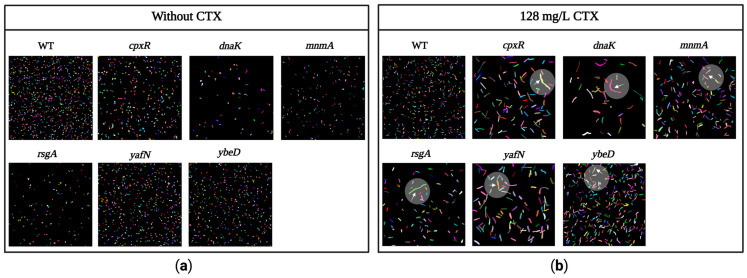
Bacterial cell morphology of WT and mutant strains in the absence (**a**) and presence of 128 mg/L CTX (**b**). Single *E. coli* cells in one microscope field of view with a magnification of 40× were artificially given a color to enhance the details of the picture. Cells in the highlighted areas with arrows show examples of cells with clear filamentation after 1 h exposure to CTX.

**Figure 6:**
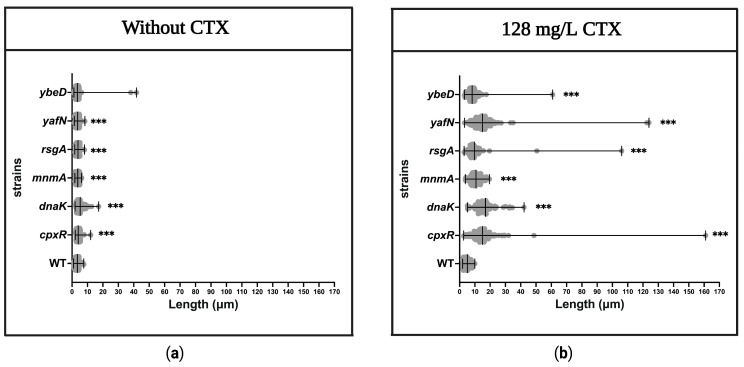
Cell length of WT and mutant strains with and without exposure to 128 mg/L CTX. Fixed cells from two biological replicates for each condition were used to measure the cell length distribution in the absence (**a**) and presence of CTX (**b**). *p*-values < 0.001 were presented as (***).

The microscopic analysis showed no obvious morphological changes in the mutant strains compared to the WT strain in the absence of CTX (Figure 5). In statistical terms, however, the average cell lengths for all the mutants, except *ΔybeD*, were significantly higher compared to the WT, with the difference in average length being between 0.16–1.79 µm (Figure 6). On the contrary, longer filament formations were clearly seen in the mutants compared to the WT in the presence of 128 mg/L CTX (Figure 5), and the average cell length for all mutants was significantly larger than the WT in the presence of CTX with increases ranging from 3.2 to 11.9 µm (Figure 6).

### 3.10. CTX Efficacy for Mutants Showing Reduced MIC to CTX

**Figure 7:**
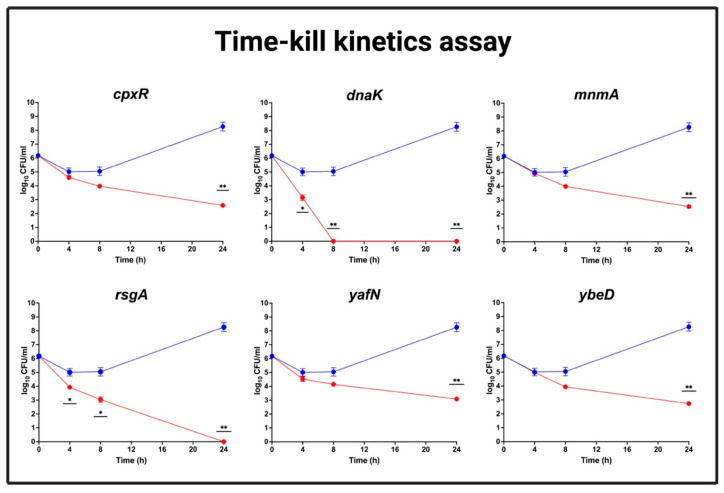
Time-kill kinetics assays for mutant strains showing reduced MIC to CTX. The activity of CTX against WT (blue) and mutant strains (red) was determined by calculating the CFU/mL at different time points (0 h, 4 h, 8 h, and 24 h) in the presence of 128 mg/L CTX. The data shown represent the mean of log10 CFU/mL with standard deviations (error bars). *p*-values < 0.05 are shown as (*) and <0.01 are shown as (**).

The reduction of MIC to CTX associated with mutations of *cpxR*, *dnaK*, *mnmA*, *rsgA*, *yafN*, and *ybeD* could be caused by either an increased efficacy of CTX or by a prolongation of the lag phase in the presence of CTX, which could limit their potential as helper drug targets. Therefore, a time-kill kinetics assays were used to evaluate the activity of CTX against these mutants and the WT in the presence of 128 mg/L CTX, which corresponds to ½ MIC of the WT (Figure 7). The analysis showed that the CFU/mL of all these mutants was significantly reduced compared to WT strain within 24 h in the presence of 128 mg/L CTX. In addition, strains with mutation of *dnaK* and *rsgA* were completely killed within 8 h and 24 h, respectively, in the presence of CTX.

### 3.11. bla_CTX-M-1_ mRNA Levels in the Mutants Identified to Increase Efficacy of CTX

**Figure 8:**
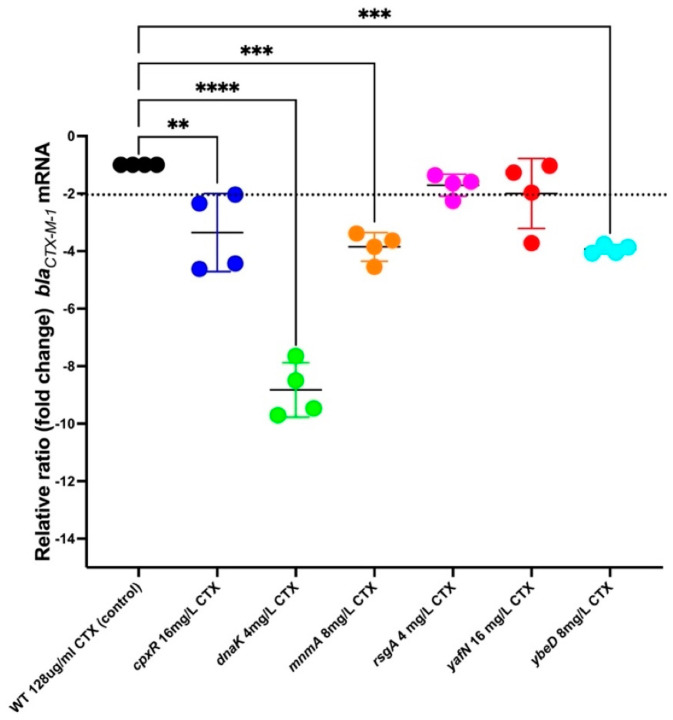
Relative changes in *bla*_CTX-M-1_ mRNA levels in the mutant strains for genes whose deletions were found to increase susceptibility to CTX. A total of two independent biological replicates, including two technical replicates, were performed. Each dot represents one sample value, and error bars represent mean with standard deviation. The data were normalized to a validated reference gene in *E. coli*: *gapA* and were relative to the *bla*_CTX-M-1_ mRNA for the WT strain in the presence of ½ MIC of CTX. ** *p* < 0.01, *** *p* < 0.001, and **** *p* < 0.0001 indicate significant differences between the control (WT) and mutants.

To investigate whether the mutant strains that were found to revitalize CTX affected the expression of *bla*_CTX-M-1_ in the presence of CTX, we exposed each mutant strain to CTX at a concentration of ½ MIC and measured the relative expression ratio of *bla*_CTX-M-1_ for the WT strain in the presence of ½ MIC. The expression analysis showed that the *bla*_CTX-M-1_ expression in all the mutant strains, except in mutations of *rsgA* and *yafN*, were significantly down-regulated relative to the WT strain in the presence of CTX. The most significant down-regulation of *bla*_CTX-M-1_ was found in mutant strain with deletion of *dnaK*, *ybeD*, *mnmA*, and *cpxR*, respectively (Figure 8). Considering the filament formations observed in these mutants upon the exposure to 128 mg/L CTX (Figure 5), the comparisons of CFU/mL between the WT and mutant strains in the presence of different CTX concentrations, which correspond to their ½ MIC, at an OD_600_ of 0.5 were calculated to make sure that roughly the same number of cells were compared in the RT-qPCR (Figure S7). The analysis showed no significant change in CFU/mL between WT and mutant strains. Therefore, this suggests that the numbers of cells used in the qPCR were comparable.

### 3.12. Homology to Human Proteome

To evaluate if the identified targets could be a promising candidate for helper drug development, the protein sequence of CpxR, YafN, RsgA, YbeD, DnaK, and MnmA were compared to human proteome using NCBI-BLAST tools to minimize unwanted side effects on the host. In silico analysis did not identify any homology of CpxR, YafN, RsgA, and YbeD from MG1655/pTF2 to human protein, whereas DnaK and MnmA were found to share homology to human 70 kilodalton (kDa) heat shock proteins (HSPs) and mitochondrial tRNA-specific 2-thiouridylase 1, respectively (Table S10).

## 4. Discussion

Although combination treatments have been used as a strategy to fight infections caused by resistant bacteria, the understanding of molecular mechanisms of the enhanced effect between antibiotics remains elusive. Since protein synthesis genes are up-regulated in the presence of CTX treatment [12], we previously hypothesized that a combination treatment with CTX and CHL would result in a synergistic effect, and indeed this was confirmed experimentally [12]. Due to the serious side effects of treatment with CHL, such as aplastic anemia [13], in the current study, we went on to evaluate the interaction between CTX and GEN, which inhibits protein synthesis by binding to the 30S ribosomal subunit instead of the 50S subunit as CHL does. This antibiotic was chosen instead of CHL because combination treatment between ß-lactams and aminoglycosides is well known and due to the fact that GEN is more relevant in human medicine [50,51,52,53]. Our results showed that the MIC of CTX could be reduced from 256 to 2 mg/L by the addition of just 0.5 mg/L GEN in *E. coli* MG1655 expressing *bla*_CTX-M-1_. Therefore, a high-throughput TraDIS screening approach was used to identify genes required for growth in the presence of CTX and GEN, and to increase our understanding of why there is synergy between these two drugs in this ESBL strain. The genes shown to be important for growth in the presence of CTX (n = 57), GEN (n = 18), and combination treatment (n = 31) constitute the SR in this strain towards these treatments, according to the definition of the SR given by Jana et al. [11].

Previously, global gene expression by RNA-seq was used to study responses in the same strain to treatment with CTX. This revealed that genes that were important for peptidoglycan biosynthesis, TCA and respiratory chain were among the most enriched functional classes [12]. In the current study, analysis of genes where insertion of transposon(s) caused significant growth attenuation confirmed the importance of the TCA cycle, and it showed that carbon metabolism was enriched in the KEGG analysis of these genes. When GO terms were analyzed, results also revealed the expected importance of peptidoglycan-based cell wall synthesis, while the protein synthesis apparatus *per se* was not suggested by the transposon analysis as important for growth in the presence of CTX. The large overlap between transcriptomic and genomic approaches gives high confidence in the results. The genomic approach used here is a more direct indicator of fitness effects, and we were able to add genes involved in cell division, bacteriocin transport, response to radiation, and outer membrane to the list of significantly enriched factors. With respect to TCA, the analysis showed that this was related to the succinate-CoA ligase and the succinate dehydrogenase complex. Previously, a similar approach was used to identify fitness genes for growth in the presence of CTX in the *bla*_CMY-23_ encoding uropathogenic *E. coli* strain EC958 of ST131. In that study, GO terms, such as cell wall organization, cell wall biogenesis, response to radiation, cell division site, outer membrane-bounded periplasmic space, peptidoglycan-based cell wall, and TAT protein transport complex, were enriched after CTX treatment [54]. These terms were also enriched in the current study, suggesting a large overlap in the SR to CTX treatment, despite different classes of ESBL genes being responsible for the resistance.

The KEGG analysis of SR genes to GEN treatment showed the enrichment of oxidative phosphorylation, metabolic pathways, and taurine and hypotaurine metabolism. Moreover, the GO analysis revealed the importance of different terms related to energy metabolism, such as the F_1_F_0_-ATP synthase complex, membrane protein complex, and protein-containing complex. These findings were in agreement with previous studies showing that ATP synthase mutants were more susceptible to aminoglycosides (e.g., GEN) than WT strains in *E. coli* and *Staphylococcus aureus* [55,56]. Furthermore, the knock-out of the gene encoding the inner membrane protein HflK, which was identified in this study as an SR gene to GEN, was also found previously to restore the susceptibility to GEN in *E. coli* [57]. Therefore, we inferred that the SR to GEN identified in this study was highly precise. For SR to combination treatment of CTX and GEN, only the oxidative phosphorylation pathway was enriched in the KEGG analysis, which we suggest is related to the GEN treatment. According to the GO terms analysis, the majority of enriched terms overlapped with terms enriched after CTX or GEN monotherapy treatment. However, GO term such as tRNA wobble position uridine thiolation, which is important for translation efficiency [58], was among the terms exclusively enriched in the combination treatment. These data add to our understanding of the importance of genes involved in protein synthesis for the synergy between the CTX and GEN.

To validate whether the identified genes in the SR to combination and single drug treatment affected the susceptibility to CTX or GEN, eight genes that were either uniquely identified in the combination treatment (*mnmA*, *rsgA*, *dnaK yafN*, and *ydeI*), overlapped with GEN treatment (*yajC* and *ybeD*), or identified as common SR genes to CTX, GEN, and combination treatment (*cpxR*) were individually deleted in MG1655/pTF2. These genes were selected because they have not previously been reported to restore susceptibility to CTX or GEN upon deletion. Four mutants (*mnmA*, *rsgA*, *dnaK*, and *ybeD*) were found to have reduced MIC to both CTX and GEN compared to WT strain, two mutants (*yafN* and *cpxR*) had reduced MIC to CTX, and the remaining two mutants (*ydeI* and *yajC*) did not show reduced MIC to any tested antibiotic. Therefore, mutants with a deletion in *mnmA*, *rsgA*, *dnaK*, *yafN*, *ybeD*, and *cpxR* were further studied to evaluate their ability to increase the efficacy of CTX.

Because CTX binds to FtsI (PBP3), which is an essential protein for cell division [59], it causes filament formation in sensitive strains [47,48]. Thus, we used fixed-cell imaging to evaluate the filament formation for the mutant strains (*dnaK*, *yafN*, *cpxR*, *mnmA*, *rsgA*, and *ybeD)* in the absence and presence of 128 mg/L CTX. As expected, only minor variations in the cell length were found between the mutant strains and the WT strain when no CTX was present. However, in the presence of CTX, apparent filament formations with variations in the cell length between the mutant strains and the WT strain were identified. Whether this includes reduced binding of CTX to FtsI or reduced levels of CTX in the cell cytosol is unknown, and further studies should focus on this.

In agreement with the microscopic analyses, the time-kill kinetics assays showed that these mutations also caused significant reduction in CFU/mL compared to WT strain in the the presence of 128 CTX. Consequently, we infer that by these mutations, we were able to increase the efficacy of CTX in the strains. As this could be attributed to either a direct effect on the *bla*_CTX-M-1_ by lowering its expression, or an indirect effect such as a disruption in the cell wall synthesis, we went on to determine whether there was an effect on the expression of the resistance. This showed that expression of *bla*_CTX-M-1_ was reduced in the *dnaK*, *ybeD*, *mnmA*, and *cpxR* mutant strains compared to the WT strain. Therefore, the re-sensitization towards CTX associated with these mutant strains might be linked to a direct effect on the *bla*_CTX-M-1_ expression, allowing a higher build-up of drug in the cell and hence the higher effect on cell wall synthesis, as illustrated by the microscopy analysis.

Some of the mutations that changed the susceptibility to CTX caused slower growth phenotype in the absence of antibiotic (*dnaK*, *mnmA*, *rsgA*, and *ybeD*) while others did not have any growth effect (*cpxR* and *yafN*). Using the latter proteins as helper drug targets is preferrable since this would not have an effect on the growth of the normal *E. coli* population. Most of the genes are well characterized, and based on current knowledge and the results observed in the study, it is possible to suggest likely mechanisms for their effect on CTX and CTX+GEN susceptibility (see Supplementary discussion S1). However, no proof of such mechanisms was performed in relation to the current study. Studies of gene-expression in the presence of the challenges in both WT strain and mutant strains would possibly enlarge our understanding of the reasons behind the observed phenotypes, and multiple methodological approaches are available for such studies [60].

Combining CTX with low concentrations of a protein-synthesis-inhibitor-antibiotic is a known principle. The synergy between such drugs was previously hypothesized to be caused by the effect of protein-synthesis-inhibitor-antibiotics (in that study CHL) on protein synthesis [12]. In the current study, we provide support for this hypothesis by identifying a high number of genes involved in protein-synthesis in the SR to combination treatment with CTX and GEN. The mutations of these genes, such as *mnmA* and *rsgA*, which are involved in translation, ribosomal structure and biogenesis, were found not only to increase the efficacy of CTX and GEN but also to increase the synergy between CTX and GEN, i.e., causing synergy at lower doses. Mutation of the gene encoding the hypothetical protein YbeD also increased the synergy between CTX and GEN. However, for this protein, the reason for its effect is unknown.

In summary, our results show that the added effect of combining CTX treatment with tre the aminoglycoside GEN is most likely linked to the effect of GEN on the protein synthesis, as we found that the interference with genes involved in protein synthesis restored the susceptibility to CTX. The study also revealed that several genes in the SR to CTX and to CTX combination treatment are promising targets for helper drugs to restore susceptibility to CTX and, for some of them, also other cephalosporin drugs.

## Figures and Tables

**Table 1:** Mapping of Tn5 insertions to K-12 MG1655 reference genome.

Library	Total Reads	Reads Mapped (%) ^1^	Total UIS ^2^	Total Seq Len/Total UIS
Input				
MG1655_pTF2_input_1	10,795,077	90.79	234,898	19.69
MG1655_pTF2_input_2	11,001,585	85.73	279,308	16.56
Input 1 + Input 2 (combined)	21,796,662	88.23	315,925	14.64
Output				
MG1655_pTF2_without_antibiotic_1	11,238,753	89.03	229,617	20.14
MG1655_pTF2_without_antibiotic_2	12,879,322	89.20	233,968	19.77
MG1655_pTF2_128mg_CTX_1	11,456,775	88.42	219,223	21.10
MG1655_pTF2_128mg_CTX_2	9,862,543	85.08	227,102	20.37
MG1655_pTF2_0.5mg_GEN_1	11,632,252	84.38	224,341	20.61
MG1655_pTF2_0.5mg_GEN_2	9,398,550	89.74	205,101	22.55
MG1655_pTF2_CTX+GEN_1	12,080,082	92.03	194,803	23.74
MG1655_pTF2_CTX+GEN_2	9,614,653	89.80	194,408	23.79

^1^ Percentage of mapped sequence reads against K-12 MG1655 reference genome. ^2^ Unique Insertion Sites.

**Table 2:** Top 20 genes according to fitness defect in the SR to CTX in *E. coli* MG1655/pTF2.

Gene	Function	128 mg/L CTX	
		Log_2_FC	q.Value
*lpoB*	penicillin-binding protein activator LpoB	−10.10	2.31 × 10^−17^
*gatB*	PTS galactitol transporter subunit IIB	−8.85	1.40 × 10^−11^
*minE*	cell division topological specificity factor MinE	−7.99	3.06 × 10^−8^
*tolQ*	Tol-Pal system protein TolQ	−7.33	6.65 × 10^−23^
*pal*	peptidoglycan-associated lipoprotein Pal	−6.96	1.63 × 10^−22^
*tolA*	cell envelope integrity protein TolA	−6.92	8.42 × 10^−19^
*tolB*	Tol-Pal system protein TolB	−6.62	1.08 × 10^−23^
*mrcB*	bifunctional glycosyl transferase/transpeptidase	−6.28	1.40 × 10^−80^
*dicC*	dicB transcriptional regulator DicC	−5.88	0.00303
*tolR*	colicin uptake protein TolR	−5.38	1.04 × 10^−27^
*rlmH*	23S rRNA (pseudouridine(1915)-N(3))-methyltransferase RlmH	−4.89	7.91 × 10^−05^
*sdhC*	succinate dehydrogenase cytochrome b556 subunit	−4.86	2.88 × 10^−10^
*ldcA*	muramoyltetrapeptide carboxypeptidase	−4.83	9.77 × 10^−14^
*sucD*	succinate--CoA ligase subunit alpha	−4.68	5.13 × 10^−22^
*plsX*	phosphate acyltransferase PlsX	−4.48	4.72 × 10^−50^
*dapB*	4-hydroxy-tetrahydrodipicolinate reductase	−4.30	0.0005
*dacA*	D-alanyl-D-alanine carboxypeptidase DacA	−4.14	2.22 × 10^−23^
*rfaD/hldD*	ADP-glyceromanno-heptose 6-epimerase	−4.13	1.31 × 10^−5^
*rsmH*	16S rRNA (cytosine(1402)-N(4))-methyltransferase RsmH	−3.91	2.52 × 10^−6^
*sltY*	murein transglycosylase	−3.88	5.43 × 10^−103^

Genes where growth was significantly affected by transposon insertions (Log_2_FC ≤ −2 and q.value ≤ 0.01) in the presence of 128 mg/L CTX. The differences in transposon insertion abundance compared to the control (without antibiotic) are shown as Log_2_FC. The false discovery risk is indicated by the q.value. The full list of genes identified as SR to CTX is provided in Table S4.

**Table 3:** Top 20 genes according to fitness defect in the SR to GEN in *E. coli* MG1655/pTF2.

Gene	Function	0.5 mg/L GEN	
		Log_2_FC	q.Value
*atpF*	F0F1 ATP synthase subunit B	−8.30	2.65 × 10^−16^
*atpG*	F0F1 ATP synthase subunit gamma	−6.49	1.19 × 10^−23^
*ybeD*	YbeD family protein	−5.60	0.005
*atpD*	F0F1 ATP synthase subunit beta	−5.55	6.69 × 10^−41^
*atpH*	F0F1 ATP synthase subunit delta	−5.50	0.009
*plsC*	1-acylglycerol-3-phosphate O-acyltransferase	−5.48	0.010
*atpC*	F0F1 ATP synthase subunit epsilon	−4.87	5.94 × 10^−11^
*atpA*	F0F1 ATP synthase subunit alpha	−4.06	1.70 × 10^−48^
*atpB*	F0F1 ATP synthase subunit A	−3.99	0.0003
*yajC*	preprotein translocase subunit YajC	−3.84	5.57 × 10^−8^
*gmhB*	D-glycero-beta-D-manno-heptose 1,7-bisphosphate 7-phosphatase	−3.22	5.11 × 10^−6^
*sdaC*	HAAAP family serine/threonine permease SdaC	−3.03	8.89 × 10^−148^
*hflC*	protease modulator HflC	−2.86	2.14 × 10^−155^
*ackA*	acetate kinase	−2.79	2.53 × 10^−9^
*cpxR*	envelope stress response regulator transcription factor CpxR	−2.66	1.40 × 10^−134^
*galU*	UTP--glucose-1-phosphate uridylyltransferase GalU	−2.57	6.36 × 10^−16^
*hflK*	FtsH protease activity modulator HflK	−2.55	8.42 × 10^−133^
*pta*	phosphate acetyltransferase	−2.02	5.07 × 10^−16^

Genes where growth was significantly affected by transposon insertions (Log_2_FC ≤ −2 and q.value ≤ 0.01) in the presence of 0.5 mg/L GEN. The differences in transposon insertion abundance compared to the control (without antibiotic) are shown as Log_2_FC. The false discovery risk is indicated by the q.value.

**Table 4:** The genes significantly depleted during combination treatment of *E. coli* MG1655/pTF2 with CTX and GEN, including illustration of the overlapping SR genes between CTX or GEN monotreatments and the combination treatment.

Gene	Function	1 + 0.25 mg/L CTX+GEN		128 mg/L CTX		0.5 mg/L GEN	
		Log_2_FC	q.Value	Log_2_FC	q.Value	Log_2_FC	q.Value
*atpD*	F0F1 ATP synthase subunit beta	−10.12	1.90 × 10^−40^	0.11	0.87	−5.55	6.69 × 10^−41^
*atpG*	F0F1 ATP synthase subunit gamma	−8.97	5.70 × 10^−23^	−0.27	0.74	−6.49	1.19 × 10^−23^
*atpF*	F0F1 ATP synthase subunit B	−8.36	1.25 × 10^−15^	1.21	0.04	−8.30	2.65 × 10^−16^
*atpC*	F0F1 ATP synthase subunit epsilon	−8.21	1.54 × 10^−12^	0.05	0.98	−4.87	5.94 × 10^−11^
*yajC*	preprotein translocase subunit YajC	−7.71	1.22 × 10^−10^	1.48	0.01	−3.84	5.57 × 10^−8^
*atpB*	F0F1 ATP synthase subunit A	−6.47	5.39 × 10^−5^	2.58	7.83 × 10^−5^	−3.99	0.0003
*mnmA*	tRNA 2-thiouridine(34) synthase MnmA	−6.04	0.0009	1.98	0.012	−0.14	0.94
*rsgA*	small ribosomal subunit biogenesis GTPase RsgA	−5.86	0.002	1.23	0.15	0.24	0.94
*ybeD*	YbeD family protein	−5.65	0.006	0.83	0.41	−5.60	0.005
*atpH*	F0F1 ATP synthase subunit delta	−5.55	0.006	2.49	0.01	−5.50	0.009
*IEU92_RS07640*	Pseudogene	−5.54	0.010	3.31	0.0003	−0.33	0.81
*ackA*	acetate kinase	−3.98	3.69 × 10^−11^	5.49	2.02 × 10^−98^	−2.79	2.53 × 10^-9^
*tolA*	cell envelope integrity protein TolA	−3.90	9.54 × 10^−33^	−6.92	8.42 × 10^−19^	0.2	0.48
*tolB*	Tol-Pal system protein TolB	−3.75	1.30 × 10^−36^	−6.62	1.08 × 10^−23^	0.13	0.59
*sdaC*	HAAAP family serine/threonine permease SdaC	−3.73	2.00 × 10^−138^	0.03	0.88	−3.03	8.89 × 10^−148^
*pal*	peptidoglycan-associated lipoprotein Pal	−3.66	3.35 × 10^−27^	−6.96	1.63 × 10^−22^	0.27	0.2
*atpA*	F0F1 ATP synthase subunit alpha	−3.64	1.47 × 10^−15^	0.77	0.08	−4.06	1.70 × 10^−48^
*IEU92_RS06395*	septation protein A	−3.49	0.010	1.91	0.04	−1.05	0.28
*yafN*	type I toxin-antitoxin system antitoxin YafN	−3.44	1.94 × 10^−5^	1.06	0.14	−0.42	0.56
*hflC*	protease modulator HflC	−3.43	1.78 × 10^−102^	−0.61	0.0008	−2.86	2.14 × 10^−155^
*tolQ*	Tol-Pal system protein TolQ	−3.36	3.31 × 10^−35^	−7.33	6.65 × 10^−23^	0.1	0.65
*cpxR*	envelope stress response regulator transcription factor	−3.33	1.14 × 10^−177^	−2.24	3.62×10^−20^	−2.66	1.40 × 10^−134^
*ydeI*	YdeI family stress tolerance OB fold protein	−3.12	3.90 × 10^−5^	−0.86	0.33	−0.58	0.37
*tusD*	sulfurtransferase complex subunit TusD	−3.05	0.0005	3.75	3.03 × 10^−10^	−1.28	0.06
*rsmH*	16S rRNA (cytosine(1402)-N(4))-methyltransferase	−3.01	8.78 × 10^−9^	−3.91	2.52 × 10^−6^	−1.62	0.0002
*dnaK*	molecular chaperone DnaK	−2.95	8.72 × 10^−5^	3.41	1.98 × 10^−9^	0.98	0.02
*tolR*	colicin uptake protein TolR	−2.89	2.37 × 10^−53^	−5.38	1.04 × 10^−27^	0.29	0.04
*hflK*	FtsH protease activity modulator HflK	−2.88	5.39 × 10^−173^	−0.08	0.62	−2.55	8.42 × 10^−133^
*pflA*	pyruvate formate lyase 1-activating protein	−2.36	2.19 × 10^−57^	1.33	7.91 × 10^−12^	−1.94	9.74 × 10^−47^
*epmA*	elongation factor P--(R)-beta-lysine ligase	−2.18	1.88 × 10^−8^	1.17	0.0002	−1.47	2.69 × 10^−13^
*pta*	phosphate acetyltransferase	−2.17	1.39 × 10^−12^	4.2	1.70 × 10^−99^	−2.02	5.07 × 10^−16^

Genes were significantly affected by transposon insertions (Log_2_FC ≤ −2 and q.value ≤ 0.01) when the input library was incubated in the presence of 1 mg/L CTX with 0.25 mg/L GEN. The differences in transposon insertion abundance compared to the control (without antibiotic) are shown as log_2_FC. The false discovery risk is indicated by the q.value. The genes shown in bold were selected for validation by site-specific knockout followed by characterization of growth in the presence of the combination treatment. The genes highlighted in green were exclusively identified as fitness genes when the input library was exposed to the combination treatment, the genes in blue and red were also significantly affected when the input library was exposed to CTX or GEN monotreatment, respectively, the genes highlighted in grey is fitness genes common to all conditions tested.

**Table 5:** MICs of CTX, GEN, and other generation cephalosporins in the mutant strains.

Strain	CTX (mg/L)	GEN (mg/L)	CFZ (mg/L)	FOX (mg/L)	CAZ (mg/L)	FEP (mg/L)	CPT (mg/L)
MG1655/pTF2 (WT)	256	1	1024	4–8	2	2	1024
*ΔcpxR*	32	0.5–1	512	4	2	2	1024
*ΔdnaK*	8	0.5	32	0.5	0.25	1	256
*ΔmnmA*	16	0.5	256	2	0.25	0.25	512
*ΔrgsA*	8	0.5	128	0.5	0.125	0.06	512
*ΔyafN*	32	1–2	512	2	2	2	1024
*ΔyajC*	128–256	0.5–1	-	-	-	-	-
*ΔydeI*	256	1–2	-	-	-	-	-
*ΔybeD*	16	0.25	512	2	0.25	0.5	1024

Values in bold represent a reduction in MIC compared to WT MG1655/pTF2. -: represents mutants that do not change the MIC to CTX.

## Data Availability

The data presented in this study are available in the article and supplementary materials.

## Supplementary Materials

The following supporting information can be downloaded at: https://www.mdpi.com/article/10.3390/antibiotics12060993/s1, Table S1: Strains and plasmids; Table S2: DNA quality and quantity of libraries; Table S3: Primers used for TraDIS; Table S4: Complete output from R scripts; Table S5: Primers used for mutagenesis and qPCR; Table S6: List of predicted essential genes in MG1655/pTF2; Tables S7–S9: GO and KEGG Pathway Enrichment Analysis for genes identified as SR to CTX, GEN, and combination treatment, respectively; Table S10: Homology to human proteins; Figure S1: Interaction between CTX and GEN in MG1655/pTF2; Figure S2: Mapping of transposon insertions; Figures S3–S5: Illustration of the interactions between the genes identified as SR to CTX, GEN, and combination treatment, respectively; Figure S6: Interaction between CTX and GEN in mutant strains; Figure S7: CFU/mL for MG1655/pTF2 and mutant strains before RNA extraction; Discussion S1: Possible mechanisms for the increased susceptibility of CTX or CTX+GEN. References [61,62,63,64,65,66,67,68,69,70,71,72,73,74,75,76,77,78,79,80,81,82,83,84,85,86,87,88,89] are only cited in the supplementary materials.

## Abbreviations

CTX-M	Cefotaximase from Munich
ESBL	Extended-Spectrum β-Lactamase
CTX	Cefotaxime
GEN	Gentamicin
MIC	Minimum Inhibitory Concentration
TraDIS	Transposon Directed Insertion-site Sequencing
USD	United States dollar
WHO	World Health Organization
PBPs	Penicillin-binding proteins
CHL	Chloramphenicol
SR	Secondary resistome
LB	Lysogeny broth
Kan	Kanamycin
CFZ	Cefazolin
FOX	Cefoxitin
CAZ	Ceftazidime
FEP	Cefepime
CPT	Ceftaroline
CLSI	Clinical and Laboratory Standards Institute
CFU/ml	Colony-Forming Unit per millilitre
MHB-II	Mueller –Hinton broth II
FIC	Fractional Inhibitory Concentration
FICI	Fractional Inhibitory Concentration Index
SOC	Super Optimal broth with Catabolites repression
UISs	Unique Insertion Sites
ENA	European Nucleotide Archive
STRING	Search Tool for the Retrieval of Interacting Genes/Proteins
KEGG	Kyoto Encyclopedia of Genes and Genomes
GO	Gene Ontology
WT	Wild type
OD_600_	Optical density at 600 nm
RT-qPCR	Reverse-Transcribed-quantitative Real Time Polymerase Chain Reaction
BLAST	Basic Local Alignment Search Tool
TCA	Tricarboxylic acid
ATP	Adenosine triphosphate
kDa	kilodalton
HSPs	Heat shock proteins
ST	Sequence Type
TAT	Twin arginine translocation
