# Supplementary material for: Uncovering the Important Genetic Factors for Growth during Cefotaxime-Gentamicin Combination Treatment in blaCTX-M-1 Encoding Escherichia coli
Source: Antibiotics (Basel). 2023 Jun 1;12(6):993. doi: 10.3390/antibiotics12060993 (PMC10295648; doi:10.3390/antibiotics12060993)
Supplement: Supplementary file 1 [file antibiotics-12-00993-s001.zip › Supplementary materials/Supplementary Tables.docx]

**Supplementary materials**

- 1. **Supplementary tables**

**Table S1.** *E. coli* strains and plasmids used in the current study.

| **Strain** | **Genotype** | **Reference** |
| --- | --- | --- |
| MG1655/pTF2  ATCC® 25922  MA100  MA101  MA102  MA103  MA104  MA105  MA106  MA107  **Plasmids**  pKD4  pKD46 | *E. coli* MG1655 + bla_CTX–M–1_on IncI1 plasmid pTF2 E. coli Reference strain  MG1655 *ΔcpxR* (Kan^R^)/pTF2 (CTX^R^)  MG1655 *ΔdnaK* (Kan^R^)/pTF2 (CTX^R^)  MG1655 *ΔmnmA* (Kan^R^)/pTF2 (CTX^R^)  MG1655 *ΔrsgA* (Kan^R^)/pTF2 (CTX^R^)  MG1655 *ΔyafN* (Kan^R^)/pTF2 (CTX^R^)  MG1655 *ΔyajC* (Kan^R^)/pTF2 (CTX^R^)  MG1655 *ΔydeI* (Kan^R^)/pTF2 (CTX^R^)  MG1655 *ΔybeD* (Kan^R^)/pTF2 (CTX^R^)  rep_R6K_ γAmp^R^ FRT Kan^R^ FRT  rep_pSC101_^ts^ Gen^R^ P*_araBAD_*γβ *exo* | [[1](#_ENREF_1)]  [[2](#_ENREF_2)]  This study  This study  This study  This study  This study  This study  This study  This study  [[3](#_ENREF_3)]  [[4](#_ENREF_4)] |

Kan^R^, CTX^R^, Amp^R^, and Gen^R^: kanamycin, cefotaxime, ampicillin, and gentamicin resistant, respectively.

| **Condition** | **T0**  **CFU/ml** | **T24 CFU/ml** | **260/280 (Nanodrop)** | **260/230 (Nanodrop)** | **Qbit HS^1^ ng/μl** |
| --- | --- | --- | --- | --- | --- |
| MG1655/pFT2_1 (input 1) | 2 × 10^9^ | - | 1.83 | 2.09 | 164 |
| MG1655/pFT2_without_antibiotic_1 | 3 × 10^6^ | 1.6 × 10^9^ | 1.86 | 2.02 | 187 |
| MG1655/pFT2_128mg_CTX_1 | 1.6 × 10^6^ | 1 × 10^9^ | 1.85 | 2.00 | 96 |
| MG1655/pFT2_0.5mg_GEN_1 | 4 × 10^6^ | 6.5 × 10^8^ | 1.99 | 2.13 | 166 |
| MG1655/pFT2_CTX+GEN_1 | 3.5 × 10^6^ | 6 × 10^8^ | 2.00 | 2.2 | 154 |
|  |  |  |  |  |  |
| MG1655/pFT2_2 (input 2) | 2 × 10^9^ | - | 1.85 | 2.17 | 220 |
| MG1655/pFT2_without_antibiotic_2 | 6.6 10^6^ | 1 × 10^9^ | 1.86 | 2.2 | 140 |
| MG1655/pFT2_128mg _CTX_2 | 3 × 10^6^ | 8 × 10^8^ | 1.85 | 2.00 | 122 |
| MG1655/pFT2_0.5mg_GEN_2 | 3.5 × 10^6^ | 4.5 × 10^8^ | 1.92 | 2.18 | 186 |
| MG1655/pFT2_CTX+GEN_2 | 3 × 10^6^ | 4 × 10^8^ | 1.95 | 2.09 | 134 |
|  |  |  |  |  |  |

**Table S2.** DNA quality and quantity of input and output libraries.

^1^dsDNA HS (High Sensitivity) Assay Kit.

**Table S3**. Primers used for TraDIS.

| **Primers^1^** | **Sequence (5’-3’)** | **Reference** |
| --- | --- | --- |
| SplA5-Top | G*AGATCGGTCTCGGCATTCCTGCTGAACCGCTCTTCCGATC*T | [[5](#_ENREF_5)] |
| SplA5-Bottom | /5PHOS/G*ATCGGAAGAGCGGTTCAGCAGGTTTTTTTTTTCAAAAAAA*A | [[5](#_ENREF_5)] |
| **Primers^2^** | **Sequence (5’-3’)** | **Reference** |
| SplAP5.1 | C*AAGCAGAAGACGGCATACGAGATAACGTGATGAGATCGGTCTCGGCATTC*C | [[5](#_ENREF_5)] |
| SplAP5.2 | C*AAGCAGAAGACGGCATACGAGATAAACATCGGAGATCGGTCTCGGCATTC*C | [[5](#_ENREF_5)] |
| SplAP5.3 | C*AAGCAGAAGACGGCATACGAGATATGCCTAAGAGATCGGTCTCGGCATTC*C | [[5](#_ENREF_5)] |
| SplAP5.4 | C*AAGCAGAAGACGGCATACGAGATAGTGGTCAGAGATCGGTCTCGGCATTC*C | [[5](#_ENREF_5)] |
| SplAP5.5 | C*AAGCAGAAGACGGCATACGAGATACCACTGTGAGATCGGTCTCGGCATTC*C | [[5](#_ENREF_5)] |
| SplAP5.6 | C*AAGCAGAAGACGGCATACGAGATACATTGGCGAGATCGGTCTCGGCATTC*C | [[5](#_ENREF_5)] |
| SplAP5.7 | C*AAGCAGAAGACGGCATACGAGATCAGATCTGGAGATCGGTCTCGGCATTC*C | [[5](#_ENREF_5)] |
| SplAP5.8 | C*AAGCAGAAGACGGCATACGAGATCATCAAGTGAGATCGGTCTCGGCATTC*C | [[5](#_ENREF_5)] |
| SplAP5.9 | C*AAGCAGAAGACGGCATACGAGATCGCTGATCGAGATCGGTCTCGGCATTC*C | [[5](#_ENREF_5)] |
| SplAP5.10 | C*AAGCAGAAGACGGCATACGAGATACAAGCTAGAGATCGGTCTCGGCATTC*C | [[5](#_ENREF_5)] |
| Tn-specific primer | AATGATACGGCGACCACCGAGATCTACACCTGATCTAGAGTCGACCTGCAGGCA TGCAAGCTTCAG | [[5](#_ENREF_5)] |
| **Primer^3^** | **Sequence (5’-3’)** | **Reference** |
| qPCR2.1 (P5) | AATGATACGGCGACCACCGAG | [[5](#_ENREF_5)] |
| qPCR 2.2 (P7) | CAAGCAGAAGACGGCATACGA | [[5](#_ENREF_5)] |
| Tn-seq-primer5 | AGGCATGCAAGCTTCAGGGTTGAGATGTGTA | [[6](#_ENREF_6)] |
| **Primer^4^** | **Sequence (5’-3’)** | **Reference** |
| iPCRtagSeq | AAGAGCGGTTCAGCAGGAATGCCGAGACCGATCTC | [[5](#_ENREF_5)] |
| Illumina Read 1 | CGGTCTCGGCATTCCTGCTGAACCGCTCTTCCGATCT | [[5](#_ENREF_5)] |
| Tn-seq-primer5 | AGGCATGCAAGCTTCAGGGTTGAGATGTGTA | [[7](#_ENREF_7)] |

^1^Primers for adapter ligation

^2^Indexed adapter-specific primers (SpIA5.x) and transposon-specific primer for PCR enrichment

^3^Primers for qPCR TraDIS libraries quantification

^4^Primers for MiSeq Illumina Sequencing

^5^Primer specific sequencing of the Tn-insertion sites. * Indicates a phosphorothioate group

**Table S4.** Complete output from R scripts showing the log_2_FC and q value of each gene (**Excel dataset**).

**Table S5.** Primers used for site-directed mutagenesis, verification of mutants, and qPCR.

| **Primer^1^** | **Sequence (5’->3’)** | **Reference** |
| --- | --- | --- |
| *cpxR_F* | CGTCTGATGACGTAATTTCTGCCTCGGAGGTATTTAAACATGTAGGCTGGAGCTGCTTC | This study |
| *cpxR_R* | AGCCAGAAGATGGCGAAGATGCGCGCGGTTAAGCTGCCTACATATGAATATCCTCCTTAG | This study |
|  |  |  |
| *dnaK_F* | ACAACCACATGATGACCGAATATATAGTGGAGACGTTTAGTGTAGGCTGGAGCTGCTTC | This study |
| *dnaK_R* | CCTTCGCCCGTGTCAGTATAATTACCCGTTTATAGGGCGACATATGAATATCCTCCTTAG | This study |
|  |  |  |
| *mnmA_F* | AGAATACGCCGCCTTGAAGTTCAATGTCGTGAGTGATCCATGTAGGCTGGAGCTGCTTC | This study |
| *mnmA_R* | GCCACGTTCACTGCTTCCTTGTTTTAAGTAAAGATAATAACATATGAATATCCTCCTTAG | This study |
|  |  |  |
| *rsgA_F* | AGATGTTTTGCCCATCAGGGGCGACCAGGAGTCTGTACGATGTAGGCTGGAGCTGCTTC | This study |
| *rsgA_R* | GAAAAAAAGGGGACGATTCTAACGACGGTTAGCTTAATTGCATATGAATATCCTCCTTAG | This study |
|  |  |  |
| *yafN_F* | ATGTATATTCTGGTGTGCATTATTATGAGGGTATCACTGTTGTAGGCTGGAGCTGCTTC | This study |
| *yafN_R* | AGTTGCAGGCGAATAAGTTTTGTTTTGAATACCCGCATCCCATATGAATATCCTCCTTAG | This study |
|  |  |  |
| *yajC_F* | CCTTTGAACGTTGATTAATATTAATAATGAGGGAAATTTATGTAGGCTGGAGCTGCTTC | This study |
| *yajC_R* | AACGGTTTAACACGGCAATTCCCTTAGGGAAAAATTTTAACATATGAATATCCTCCTTAG | This study |
|  |  |  |
| *ydel_F* | AGACTTAATAAGTCAAACCAAACGCAATTAAGGAGTTAGTTGTAGGCTGGAGCTGCTTC | This study |
| *ydel_R* | CTAATGGCCCAGTAGTTCAGGCCATCAGGCTAATTTATTTCATATGAATATCCTCCTTAG | This study |
|  |  |  |
| *ybeD_F* | GGCGGGAGTTGCTATTTAATTACGTTACGCCGGAGCTGACTGTAGGCTGGAGCTGCTTC | This study |
| *ybeD_R* | GGGAGTTACCCGACCAGGAGCCGGGTAACGGAGAAGCGAGCATATGAATATCCTCCTTAG | This study |
|  |  |  |
| **Primer^2^** | **Sequence (5’->3’)** | **Reference** |
| *cpxR_F* | GCCAGTTATCGCCTGAACCG | This study |
| *cpxR_R* | CTGGCGGTGCCCACTTATCA | This study |
|  |  |  |
| *dnaK_F* | ATATGAATAAAATTGCTGTC | This study |
| *dnaK_R* | TTGATCTCTTTAAATTTCGC | This study |
|  |  |  |
| *mnmA_F* | CCCGACCCAGCCTCATGACA | This study |
| *mnmA_R* | GGGTTTCCAGCCCGACGCGC | This study |
|  |  |  |
| *rsgA_F* | TGTACTGCAATGGTCGGCCC | This study |
| *rsgA_R* | GTCGACCTTGTAGTATTTAA | This study |
|  |  |  |
| *yafN_F* | TTAAAGTTCGACGATTTTCA | This study |
| *yafN_R* | TTCTGCTGAATTGGCGCAAC | This study |
|  |  |  |
| *yajC_F* | GCTACACCTGTCGCAATTAT | This study |
| *yajC_R* | CTCTTCCAGTGCCACAGACT | This study |
|  |  |  |
| *ydel_F* | TTGCTTTTTCTGTATGCTCG | This study |
| *ydel_R* | CACAGTTCCTTGTGCGCGCT | This study |
|  |  |  |
| *ybeD_F* | TCAACTTCCAGCTTGATGGC | This study |
| *ybeD_R* | AATACCGGATAGTGCTCGAC | This study |
|  |  |  |
| **Primer^3^** | **Sequence (5’->3’)** | **Reference** |
| *CTX-M-1-F* | GACTATGGCACCACCAACG | [[1](#_ENREF_1)] |
| *CTX-M-1-R* | GCTTTCTGCCTTAGGTTGAGG | [[1](#_ENREF_1)] |
| *gapA-F* | ACTGACTGGTATGGCGTTCC | [[1](#_ENREF_1)] |
| *gapA-R* | GTTGCAGCTTTTTCCAGACG | [[1](#_ENREF_1)] |

^1^Primers for site-directed mutagenesis, ^2^Primers for proof of insertion, and ^3^Primers for RT-qPCR

**Table S6.** List of genes essential for growth on LB agar plate supplemented with kanamycin (**Excel dataset**).

**Tables S7, S8, and S9**. GO and KEGG Pathway Enrichment Analysis for genes identified as secondary resistome to CTX, GEN, and combination treatment of CTX and GEN (**Excel dataset**).

**Table S10.** Homology of DnaK and MnmA from MG1655 to human protein (**Excel dataset**).

**References**

1. Kjeldsen, T.S.; Overgaard, M.; Nielsen, S.S.; Bortolaia, V.; Jelsbak, L.; Sommer, M.; Guardabassi, L.; Olsen, J.E. CTX-M-1 β-lactamase expression in Escherichia coli is dependent on cefotaxime concentration, growth phase and gene location. J Antimicrob Chemother 2015, 70, 62-70, doi:10.1093/jac/dku332.

2. Ceri, H.; Olson, M.E.; Stremick, C.; Read, R.R.; Morck, D.; Buret, A. The Calgary Biofilm Device: new technology for rapid determination of antibiotic susceptibilities of bacterial biofilms. J Clin Microbiol 1999, 37, 1771-1776, doi:10.1128/jcm.37.6.1771-1776.1999.

3. Datsenko, K.A.; Wanner, B.L. One-step inactivation of chromosomal genes in Escherichia coli K-12 using PCR products. Proc Natl Acad Sci U S A 2000, 97, 6640-6645, doi:10.1073/pnas.120163297.

4. Doublet, B.; Douard, G.; Targant, H.; Meunier, D.; Madec, J.Y.; Cloeckaert, A. Antibiotic marker modifications of lambda Red and FLP helper plasmids, pKD46 and pCP20, for inactivation of chromosomal genes using PCR products in multidrug-resistant strains. J Microbiol Methods 2008, 75, 359-361, doi:10.1016/j.mimet.2008.06.010.

5. Barquist, L.; Mayho, M.; Cummins, C.; Cain, A.K.; Boinett, C.J.; Page, A.J.; Langridge, G.C.; Quail, M.A.; Keane, J.A.; Parkhill, J. The TraDIS toolkit: sequencing and analysis for dense transposon mutant libraries. Bioinformatics 2016, 32, 1109-1111, doi:10.1093/bioinformatics/btw022.

6. Berg, D.E.; Weiss, A.; Crossland, L. Polarity of Tn5 insertion mutations in Escherichia coli. J Bacteriol 1980, 142, 439-446, doi:10.1128/jb.142.2.439-446.1980.

7. Meyer, C.; Hoffmann, C.; Haas, R.; Schubert, S. The role of the galU gene of uropathogenic Escherichia coli in modulating macrophage TNF-α response. Int J Med Microbiol 2015, 305, 893-901, doi:10.1016/j.ijmm.2015.09.004.
