# Supplementary material for: Uncovering the Important Genetic Factors for Growth during Cefotaxime-Gentamicin Combination Treatment in blaCTX-M-1 Encoding Escherichia coli
Source: Antibiotics (Basel). 2023 Jun 1;12(6):993. doi: 10.3390/antibiotics12060993 (PMC10295648; doi:10.3390/antibiotics12060993)
Supplement: Supplementary file 1 [file antibiotics-12-00993-s001.zip › Supplementary materials/Supplementary Figures.docx]

**Supplementary materials**

- 1. **Supplementary figures**


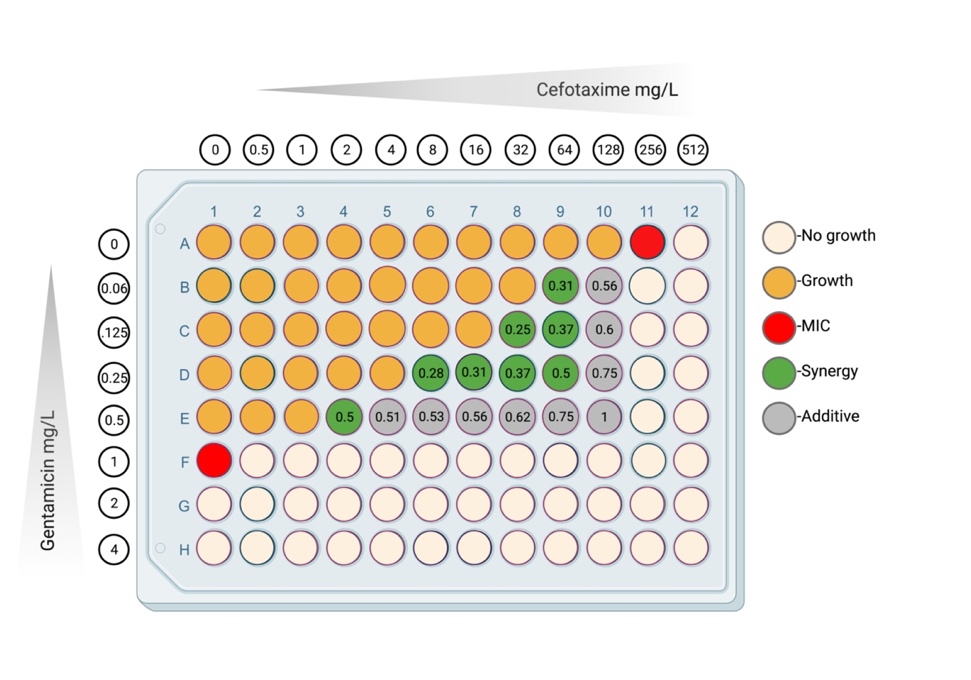


**Figure S1.** Interaction between CTX and GEN. Chequerboard assay and fractional inhibitory concentrations indexes (FICIs) for WT (MG1655/pTF2) with CTX and GEN. The MICs of CTX, GEN for MG1655/pTF2 were 256 mg/L, 1 mg/L, respectively.


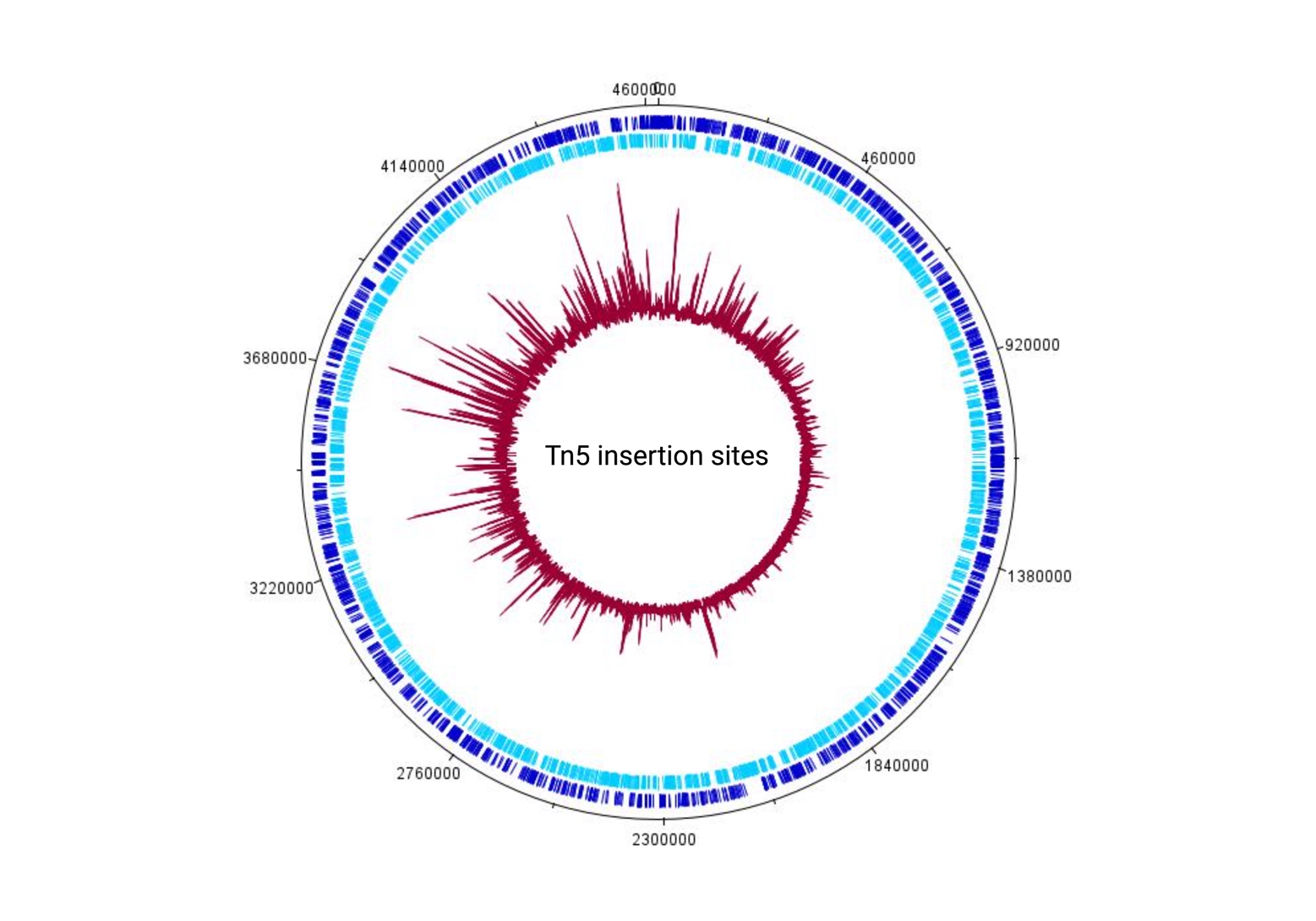


**Figure S2.** Mapping of transposon insertion sites to the reference genome of *E. coli* MG1655. The outer ring in black represents the base positions, and the sense and antisense coding sequences (CDSs) are displayed in blue and cyan, respectively. The dark red lines show the number of Tn5 insertions at each position in the genome (315,925 unique Tn5 insertion sites were identified in the input library).


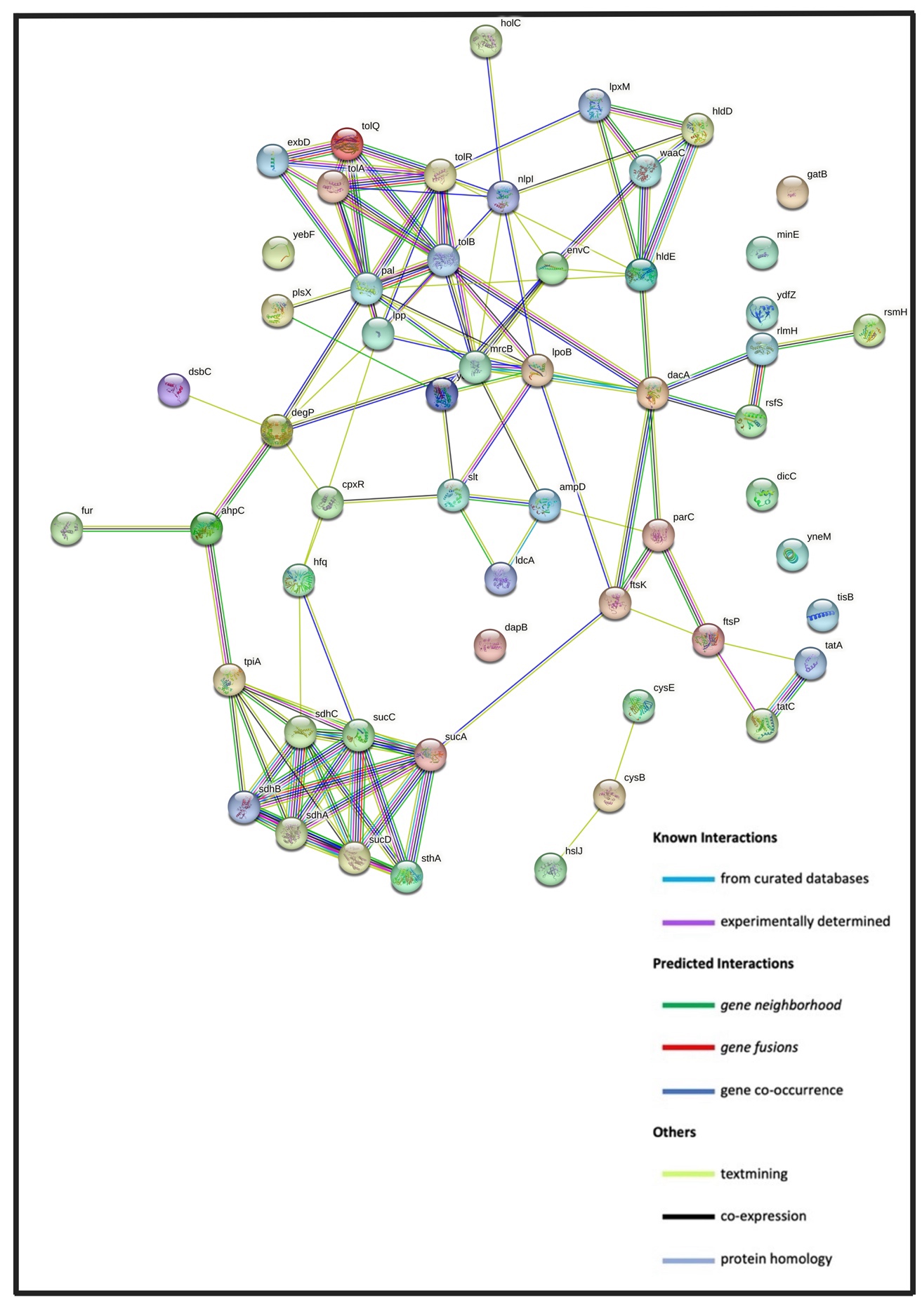


**Figure S3.** STRING analysis graphical presentation of the interactions between the genes identified as SR to CTX treatment.


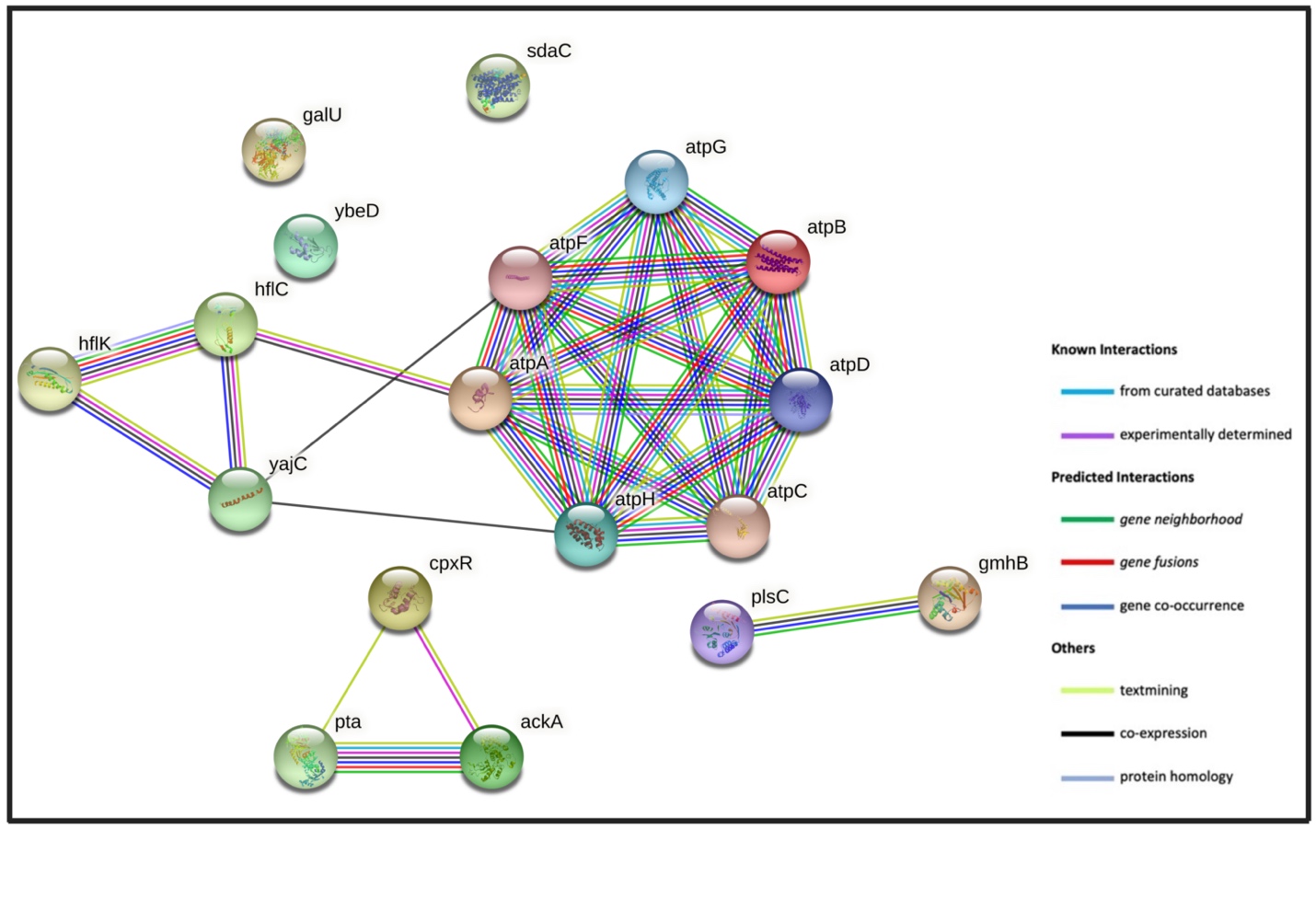


**Figure S4.** STRING analysis graphical presentation of the interactions between the genes identified as SR to GEN treatment.


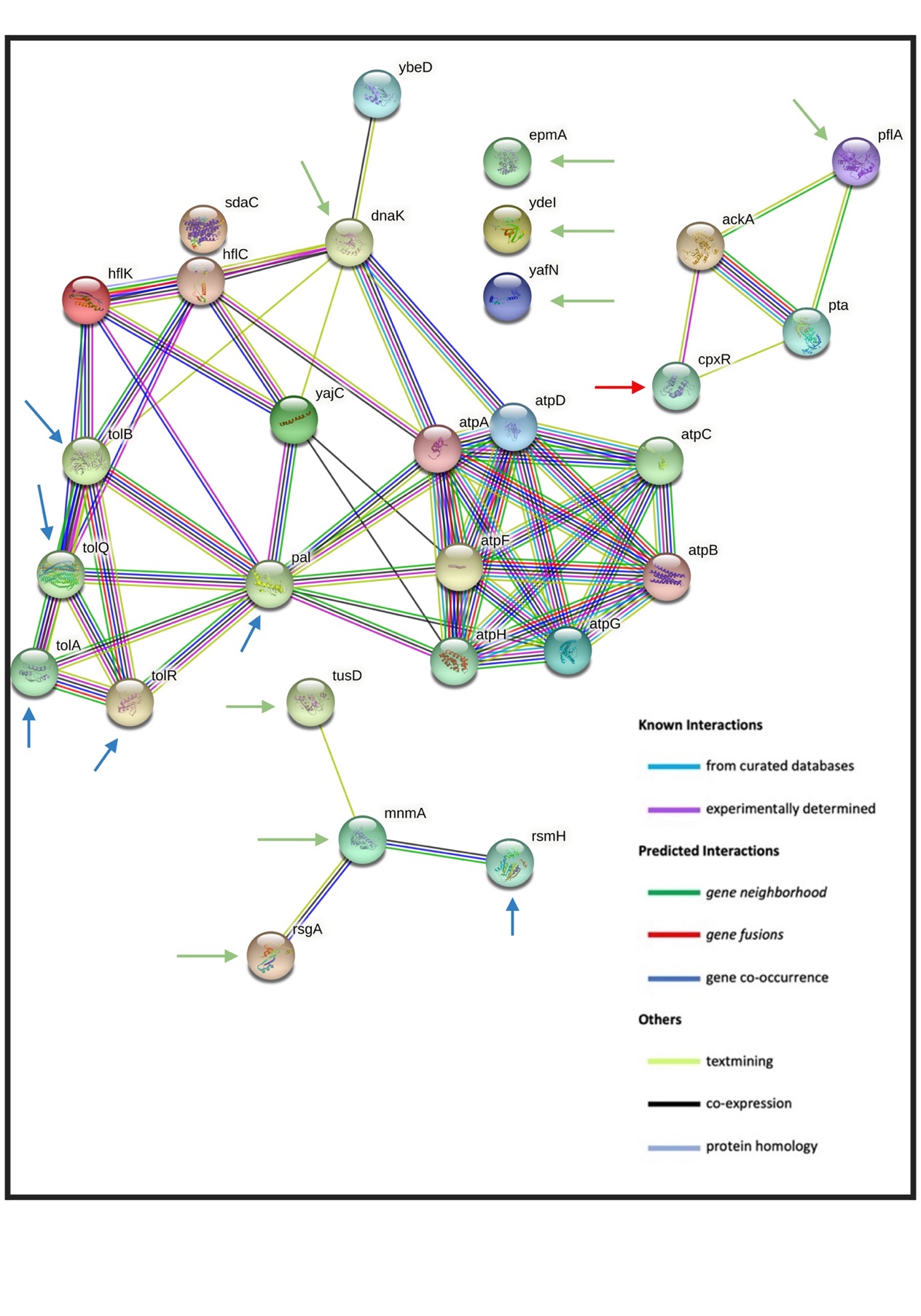


**Figure S5.** STRING analysis graphical presentation of the interactions between the genes identified as SR to combination treatments of CTX and GEN. Green arrows represent SR genes that uniquely identified in combination treatment, blue arrows represent SR genes overlapped with monotreatment of CTX, red arrows represent SR genes that are shared between combination treatment and monotherapy of CTX and GEN, and SR genes without arrows are overlapped with SR genes to GEN.


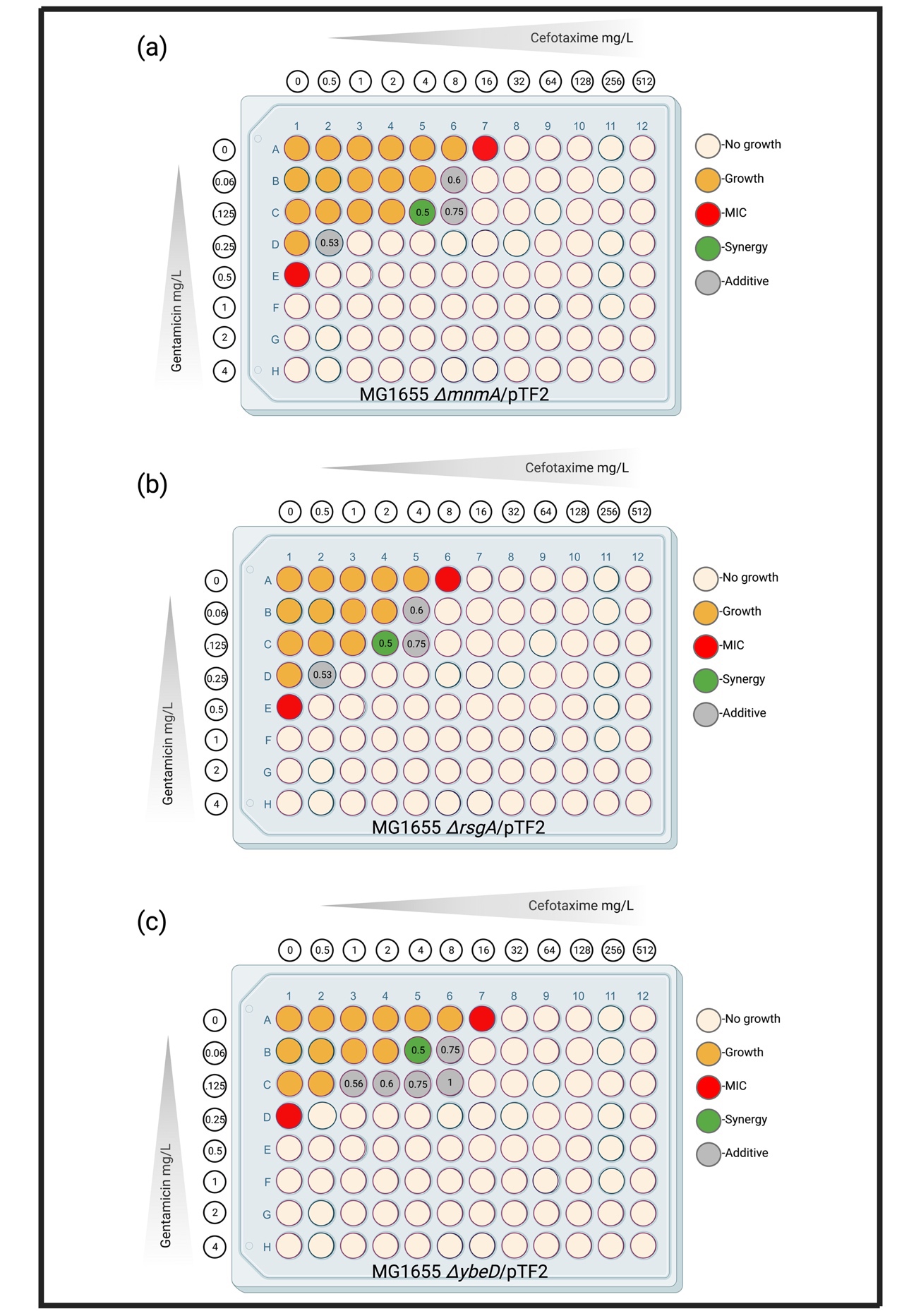


**Figure S6.** Chequerboard assays and fractional inhibitory concentrations indexes (FICIs) for (a) MG1655*ΔmnmA*/pTF2, (b) MG1655*ΔrsgA*/pTF2, and (c) MG1655*ΔybeD*/pTF2 with CTX and GEN.

**
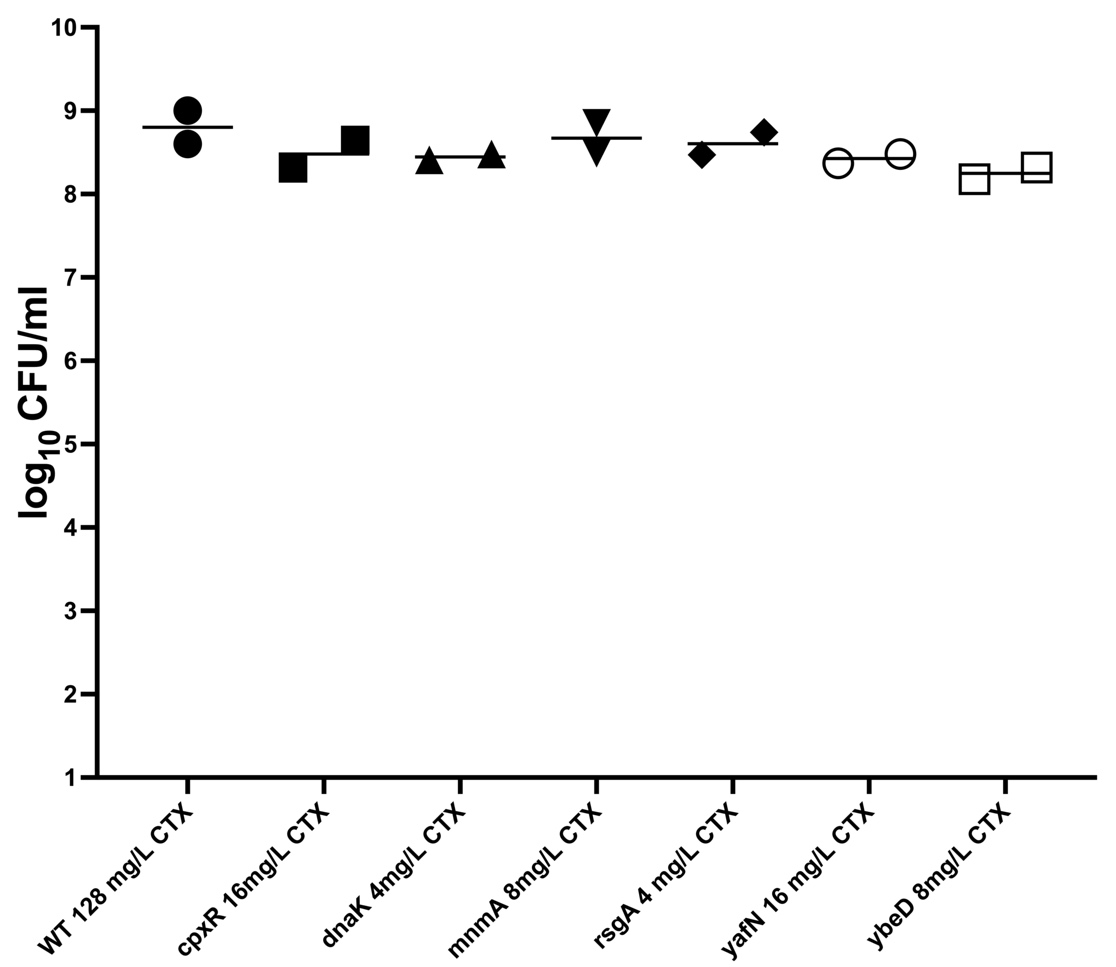
**

**Figure S7.** Graphical representation of log10 CFU/ml for MG1655/pTF2 and mutant strains before RNA extraction. The CFU/ml for each strain was determined in the presence of ½ MIC CTX at an OD_600_ of 0.5-0.6. Two independent biological replicates were performed for each strain, and the significance change compared to the WT was evaluated using a one-way ANOVA. P-values <0.05 were shown as (*).
