# Supplementary material for: Uncovering the Important Genetic Factors for Growth during Cefotaxime-Gentamicin Combination Treatment in blaCTX-M-1 Encoding Escherichia coli
Source: Antibiotics (Basel). 2023 Jun 1;12(6):993. doi: 10.3390/antibiotics12060993 (PMC10295648; doi:10.3390/antibiotics12060993)
Supplement: Supplementary file 1 [file antibiotics-12-00993-s001.zip › Supplementary materials/Supplementary Discussion.docx]

**Supplementary materials**

- 1. **Supplementary discussion**

**Supplementary discussion S1.** Possible mechanism(s) for the increased susceptibility of CTX or CTX+GEN upon the inactivation of the selected protein-coding genes in MG1655/pTF2.

Among the mutants that cause slow growth phenotype, DnaK is a heat shock protein that plays a role in different cytoplasmic cellular processes involving protein secretion [[1](#_ENREF_1),[2](#_ENREF_2)], folding newly synthesized proteins [[3](#_ENREF_3),[4](#_ENREF_4)], and rescue of misfolded proteins [[5-7](#_ENREF_5)]. Mutation of *dnaK* was found to decrease the MICs of CTX, other tested cephalosporins, and GEN. The increased sensitivity to CTX could be explained by the observed reduction in the expression of *bla_CTX-M-1_*, however, it could also be related to the involvement of DnaK in the ribosome assembly and the secretion of the SecB-independent proteins (alkaline phosphatase, ribose-binding protein, and β-lactamase) [[2](#_ENREF_2),[8](#_ENREF_8)]. Inactivation of *dnaK* was previously shown to increase the susceptibility of *Staphylococcus aureus* to oxacillin and methicillin as well as *E. coli* to fluoroquinolones [[9](#_ENREF_9),[10](#_ENREF_10)]. However, in agreement with our *in silico* results, DnaK was previously reported to share about 50% amino acid homology with eukaryotic HSP-70 proteins [[11](#_ENREF_11)]. Thus, it may not be suited as a helper drug target. Nevertheless, it is worth mentioning that HSP-70 is considered a promising anti-cancer target because it is highly expressed in multiple forms of human cancer, leading to inhibit the apoptosis of malignant cells and promote metastasis [[12](#_ENREF_12)].

MnmA is involved in the tRNA metabolic process by catalyzing the 2-thiolation of uridine at the wobble position (U34) of tRNA^Lysine^, tRNA^Glutamine^ and tRNA^Glutamate^ leading to the formation of sulfur at the 2-position (s2U34), the first step of tRNA- modified nucleoside 5-methylamino-methyl-2-thiouridine (mnm5s2U34) synthesis [[13](#_ENREF_13)]. Although a previous study claimed that *mnmA* is an essential gene [[14](#_ENREF_14)], the current study showed that deletion of the gene resulted in a slow growth phenotype, which has also been reported in other studies [[13](#_ENREF_13),[15](#_ENREF_15),[16](#_ENREF_16)]. Deletion of *mnmA* resulted in increased susceptibility to CTX, other cephalosporins, and GEN. Thiouridylase (MnmA) is important for accurate and efficient translation during protein synthesis [[17](#_ENREF_17),[18](#_ENREF_18)], and the re-sensitization of CTX in the *mnmA* mutant strain might be linked to the inefficient translation of CTX-M-1. MnmA has similarity to human mitochondrial tRNA-specific 2-thiouridylase 1 (with 63% query coverage and 43.58% identity), and therefore, it may not be a good helper drug target.

RsgA is a GTPases, which assists in the maturation of the 30S ribosomal subunit [[19-22](#_ENREF_19)]. The slow growth phenotype observed in *rsgA* mutant strain agrees with previous studies [[19](#_ENREF_19),[23](#_ENREF_23)]. Deletion of this gene caused increased efficacy of CTX and GEN without significantly affecting the expression of the resistant gene. It might be the increase in CTX efficacy was due to the absence of some late ribosomal proteins and immature 30S subunits [[20](#_ENREF_20),[24](#_ENREF_24)], however, further experiments, such as measuring the CTX-M-1 protein level in the *rsgA* mutant strain compared to WT in the presence of CTX are indicated.

YbeD is a hypothetical protein located in the intergenic region of *dacA*-*lipB*, which encodes a D-alanyl-D-alanine carboxypeptidase involved in peptidoglycan biosynthesis, and the *lip* operon that includes genes responsible for lipoic acid biosynthesis [[25](#_ENREF_25)]. Moreover, YbeD was previously found to be involved in the response to high-temperature stress, where a *ybeD* mutant strain grew slower and to a lower cell density than the *E. coli* WT strain [[26](#_ENREF_26)]. Removal of *ybeD* was previously reported to increase the susceptibility to ampicillin in *E. coli* [[27](#_ENREF_27)], and as our results added CTX and GEN to the list of affected antibiotics, we suggest that YbeD may be used as a target for helper drugs for several antimicrobials.

The deletions of *yafN* and *cpxR* were found to increase the efficacy of CTX without causing a growth defect in the absence of antibiotics. Thus, their encoded proteins could be better helper drug targets than those above, since using them as targets would not have an effect on the growth of the normal *E. coli* population. YafN is an antitoxin for the ribosome-associated mRNA interferase toxin YafO, which inhibits protein synthesis [[28-30](#_ENREF_28)]. The overexpression of YafN was previously found to prevent YafO from inhibiting translation [[30](#_ENREF_30)], and several studies have reported that *yafN* is an essential gene, when *yafO* is functional [[29](#_ENREF_29),[31](#_ENREF_31)]. In our study, site-specific removal of *yafN* was not affecting the growth of the strain in the absence of antibiotics, and it may be that YafO is dys-functioning in our strain, as was previously identified in *E. coli* JW0222 [[29](#_ENREF_29),[31](#_ENREF_31)]. Further studies are required to elucidate the reason behind the increase susceptibility to CTX in this mutant strain.

Interestingly, our analysis identified *cpxR* as a gene in the SR to combination treatment as well as to monotreatment with CTX or GEN, making it an interesting target for helper drugs. CpxR is a response regulator protein of the two-component regulatory system CpxA/CpxR. This system responds to envelope stress by activating expression of downstream genes (*cpxP, degP, dsbA* and *ppiA*) involved in envelope protein folding and degrading enzymes [[32](#_ENREF_32),[33](#_ENREF_33)]. In this study, we found a reduction in the MIC of only CTX in *cpxR* mutant strain. Since the overproduction of CpxR was previously identified to confer resistance to β-lactam in a *E. coli* strain lacking *acrB* [[34](#_ENREF_34)], it is not surprising that the deletion of *cpxR* could result in increased the susceptibly to CTX.

**References**

1. Wild, J.; Altman, E.; Yura, T.; Gross, C.A. DnaK and DnaJ heat shock proteins participate in protein export in Escherichia coli. *Genes Dev* **1992**, *6*, 1165-1172, doi:10.1101/gad.6.7.1165.

2. Wild, J.; Rossmeissl, P.; Walter, W.A.; Gross, C.A. Involvement of the DnaK-DnaJ-GrpE chaperone team in protein secretion in Escherichia coli. *J Bacteriol* **1996**, *178*, 3608-3613, doi:10.1128/jb.178.12.3608-3613.1996.

3. Deuerling, E.; Schulze-Specking, A.; Tomoyasu, T.; Mogk, A.; Bukau, B. Trigger factor and DnaK cooperate in folding of newly synthesized proteins. *Nature* **1999**, *400*, 693-696, doi:10.1038/23301.

4. Teter, S.A.; Houry, W.A.; Ang, D.; Tradler, T.; Rockabrand, D.; Fischer, G.; Blum, P.; Georgopoulos, C.; Hartl, F.U. Polypeptide flux through bacterial Hsp70: DnaK cooperates with trigger factor in chaperoning nascent chains. *Cell* **1999**, *97*, 755-765, doi:10.1016/s0092-8674(00)80787-4.

5. Skowyra, D.; Georgopoulos, C.; Zylicz, M. The E. coli dnaK gene product, the hsp70 homolog, can reactivate heat-inactivated RNA polymerase in an ATP hydrolysis-dependent manner. *Cell* **1990**, *62*, 939-944, doi:10.1016/0092-8674(90)90268-j.

6. Ziemienowicz, A.; Skowyra, D.; Zeilstra-Ryalls, J.; Fayet, O.; Georgopoulos, C.; Zylicz, M. Both the Escherichia coli chaperone systems, GroEL/GroES and DnaK/DnaJ/GrpE, can reactivate heat-treated RNA polymerase. Different mechanisms for the same activity. *J Biol Chem* **1993**, *268*, 25425-25431.

7. Schröder, H.; Langer, T.; Hartl, F.U.; Bukau, B. DnaK, DnaJ and GrpE form a cellular chaperone machinery capable of repairing heat-induced protein damage. *Embo j* **1993**, *12*, 4137-4144, doi:10.1002/j.1460-2075.1993.tb06097.x.

8. Alix, J.H.; Guérin, M.F. Mutant DnaK chaperones cause ribosome assembly defects in Escherichia coli. *Proc Natl Acad Sci U S A* **1993**, *90*, 9725-9729, doi:10.1073/pnas.90.20.9725.

9. Singh, V.K.; Utaida, S.; Jackson, L.S.; Jayaswal, R.K.; Wilkinson, B.J.; Chamberlain, N.R. Role for dnaK locus in tolerance of multiple stresses in Staphylococcus aureus. *Microbiology (Reading)* **2007**, *153*, 3162-3173, doi:10.1099/mic.0.2007/009506-0.

10. Yamaguchi, Y.; Tomoyasu, T.; Takaya, A.; Morioka, M.; Yamamoto, T. Effects of disruption of heat shock genes on susceptibility of Escherichia coli to fluoroquinolones. *BMC Microbiol* **2003**, *3*, 16, doi:10.1186/1471-2180-3-16.

11. Daugaard, M.; Rohde, M.; Jäättelä, M. The heat shock protein 70 family: Highly homologous proteins with overlapping and distinct functions. *FEBS Lett* **2007**, *581*, 3702-3710, doi:10.1016/j.febslet.2007.05.039.

12. Boudesco, C.; Cause, S.; Jego, G.; Garrido, C. Hsp70: A Cancer Target Inside and Outside the Cell. *Methods Mol Biol* **2018**, *1709*, 371-396, doi:10.1007/978-1-4939-7477-1_27.

13. Kambampati, R.; Lauhon, C.T. MnmA and IscS are required for in vitro 2-thiouridine biosynthesis in Escherichia coli. *Biochemistry* **2003**, *42*, 1109-1117, doi:10.1021/bi026536+.

14. Arigoni, F.; Talabot, F.; Peitsch, M.; Edgerton, M.D.; Meldrum, E.; Allet, E.; Fish, R.; Jamotte, T.; Curchod, M.L.; Loferer, H. A genome-based approach for the identification of essential bacterial genes. *Nat Biotechnol* **1998**, *16*, 851-856, doi:10.1038/nbt0998-851.

15. Dassain, M.; Leroy, A.; Colosetti, L.; Carolé, S.; Bouché, J.P. A new essential gene of the 'minimal genome' affecting cell division. *Biochimie* **1999**, *81*, 889-895, doi:10.1016/s0300-9084(99)00207-2.

16. Nilsson, K.; Lundgren, H.K.; Hagervall, T.G.; Björk, G.R. The cysteine desulfurase IscS is required for synthesis of all five thiolated nucleosides present in tRNA from Salmonella enterica serovar typhimurium. *J Bacteriol* **2002**, *184*, 6830-6835, doi:10.1128/jb.184.24.6830-6835.2002.

17. Numata, T.; Ikeuchi, Y.; Fukai, S.; Suzuki, T.; Nureki, O. Snapshots of tRNA sulphuration via an adenylated intermediate. *Nature* **2006**, *442*, 419-424, doi:10.1038/nature04896.

18. Bimai, O.; Arragain, S.; Golinelli-Pimpaneau, B. Structure-based mechanistic insights into catalysis by tRNA thiolation enzymes. *Curr Opin Struct Biol* **2020**, *65*, 69-78, doi:10.1016/j.sbi.2020.06.002.

19. Campbell, T.L.; Brown, E.D. Genetic interaction screens with ordered overexpression and deletion clone sets implicate the Escherichia coli GTPase YjeQ in late ribosome biogenesis. *J Bacteriol* **2008**, *190*, 2537-2545, doi:10.1128/jb.01744-07.

20. Jomaa, A.; Stewart, G.; Martín-Benito, J.; Zielke, R.; Campbell, T.L.; Maddock, J.R.; Brown, E.D.; Ortega, J. Understanding ribosome assembly: the structure of in vivo assembled immature 30S subunits revealed by cryo-electron microscopy. *Rna* **2011**, *17*, 697-709, doi:10.1261/rna.2509811.

21. Jomaa, A.; Stewart, G.; Mears, J.A.; Kireeva, I.; Brown, E.D.; Ortega, J. Cryo-electron microscopy structure of the 30S subunit in complex with the YjeQ biogenesis factor. *Rna* **2011**, *17*, 2026-2038, doi:10.1261/rna.2922311.

22. López-Alonso, J.P.; Kaminishi, T.; Kikuchi, T.; Hirata, Y.; Iturrioz, I.; Dhimole, N.; Schedlbauer, A.; Hase, Y.; Goto, S.; Kurita, D.; et al. RsgA couples the maturation state of the 30S ribosomal decoding center to activation of its GTPase pocket. *Nucleic Acids Res* **2017**, *45*, 6945-6959, doi:10.1093/nar/gkx324.

23. Himeno, H.; Hanawa-Suetsugu, K.; Kimura, T.; Takagi, K.; Sugiyama, W.; Shirata, S.; Mikami, T.; Odagiri, F.; Osanai, Y.; Watanabe, D.; et al. A novel GTPase activated by the small subunit of ribosome. *Nucleic Acids Res* **2004**, *32*, 5303-5309, doi:10.1093/nar/gkh861.

24. Thurlow, B.; Davis, J.H.; Leong, V.; Moraes, T.F.; Williamson, J.R.; Ortega, J. Binding properties of YjeQ (RsgA), RbfA, RimM and Era to assembly intermediates of the 30S subunit. *Nucleic Acids Res* **2016**, *44*, 9918-9932, doi:10.1093/nar/gkw613.

25. Kozlov, G.; Elias, D.; Semesi, A.; Yee, A.; Cygler, M.; Gehring, K. Structural similarity of YbeD protein from Escherichia coli to allosteric regulatory domains. *Journal of bacteriology* **2004**, *186*, 8083-8088.

26. Kim, S.; Kim, Y.; Yoon, S.H. Overexpression of YbeD in Escherichia coli Enhances Thermotolerance. *J Microbiol Biotechnol* **2019**, *29*, 401-409, doi:10.4014/jmb.1901.01036.

27. Tamae, C.; Liu, A.; Kim, K.; Sitz, D.; Hong, J.; Becket, E.; Bui, A.; Solaimani, P.; Tran, K.P.; Yang, H.; et al. Determination of antibiotic hypersensitivity among 4,000 single-gene-knockout mutants of Escherichia coli. *J Bacteriol* **2008**, *190*, 5981-5988, doi:10.1128/jb.01982-07.

28. Zhang, Y.; Yamaguchi, Y.; Inouye, M. Characterization of YafO, an Escherichia coli toxin. *J Biol Chem* **2009**, *284*, 25522-25531, doi:10.1074/jbc.M109.036624.

29. Singletary, L.A.; Gibson, J.L.; Tanner, E.J.; McKenzie, G.J.; Lee, P.L.; Gonzalez, C.; Rosenberg, S.M. An SOS-regulated type 2 toxin-antitoxin system. *J Bacteriol* **2009**, *191*, 7456-7465, doi:10.1128/jb.00963-09.

30. Christensen-Dalsgaard, M.; Jørgensen, M.G.; Gerdes, K. Three new RelE-homologous mRNA interferases of Escherichia coli differentially induced by environmental stresses. *Mol Microbiol* **2010**, *75*, 333-348, doi:10.1111/j.1365-2958.2009.06969.x.

31. Baba, T.; Ara, T.; Hasegawa, M.; Takai, Y.; Okumura, Y.; Baba, M.; Datsenko, K.A.; Tomita, M.; Wanner, B.L.; Mori, H. Construction of Escherichia coli K-12 in-frame, single-gene knockout mutants: the Keio collection. *Mol Syst Biol* **2006**, *2*, 2006.0008, doi:10.1038/msb4100050.

32. Danese, P.N.; Snyder, W.B.; Cosma, C.L.; Davis, L.J.; Silhavy, T.J. The Cpx two-component signal transduction pathway of Escherichia coli regulates transcription of the gene specifying the stress-inducible periplasmic protease, DegP. *Genes Dev* **1995**, *9*, 387-398, doi:10.1101/gad.9.4.387.

33. Raivio, T.L.; Silhavy, T.J. Transduction of envelope stress in Escherichia coli by the Cpx two-component system. *J Bacteriol* **1997**, *179*, 7724-7733, doi:10.1128/jb.179.24.7724-7733.1997.

34. Hirakawa, H.; Nishino, K.; Yamada, J.; Hirata, T.; Yamaguchi, A. Beta-lactam resistance modulated by the overexpression of response regulators of two-component signal transduction systems in Escherichia coli. *J Antimicrob Chemother* **2003**, *52*, 576-582, doi:10.1093/jac/dkg406.
